# The overexpression of TDP-43 in astrocytes causes neurodegeneration via a PTP1B-mediated inflammatory response

**DOI:** 10.1186/s12974-020-01963-6

**Published:** 2020-10-14

**Authors:** Shinrye Lee, Seyeon Kim, Ha-Young Kang, Hye Ryeong Lim, Younghwi Kwon, Myungjin Jo, Yu-Mi Jeon, Sang Ryong Kim, Kiyoung Kim, Chang Man Ha, Seongsoo Lee, Hyung-Jun Kim

**Affiliations:** 1 Dementia Research Group, Korea Brain Research Institute (KBRI), Daegu, 41062 South Korea; 2 Department of Brain & Cognitive Sciences, DGIST, Daegu, 42988 South Korea; 3 Gwangju Center, Korea Basic Science Institute (KBSI), Gwangju, 61886 South Korea; 4 Research Division and Brain Research Core Facilities, Korea Brain Research Institute (KBRI), Daegu, 41062 South Korea; 5 School of Life Sciences, BK21 Plus KNU Creative BioResearch Group, Institute of Life Science & Biotechnology, Kyungpook National University, Daegu, 41566 South Korea; 6 Brain Science and Engineering Institute, Kyungpook National University, Daegu, 41944 South Korea; 7 Department of Medical Biotechnology, Soonchunhyang University, Asan, 31538 South Korea

**Keywords:** Neurodegenerative disease, Neuroinflammation, Astrocytes, Tar DNA-binding protein 43, Protein tyrosine phosphatase 1B

## Abstract

**Background:**

Cytoplasmic inclusions of transactive response DNA binding protein of 43 kDa (TDP-43) in neurons and astrocytes are a feature of some neurodegenerative diseases, such as frontotemporal lobar degeneration with TDP-43 (FTLD-TDP) and amyotrophic lateral sclerosis (ALS). However, the role of TDP-43 in astrocyte pathology remains largely unknown.

**Methods:**

To investigate whether TDP-43 overexpression in primary astrocytes could induce inflammation, we transfected primary astrocytes with plasmids encoding *Gfp* or *TDP*-*43*-*Gfp*. The inflammatory response and upregulation of PTP1B in transfected cells were examined using quantitative RT-PCR and immunoblot analysis. Neurotoxicity was analysed in a transwell coculture system of primary cortical neurons with astrocytes and cultured neurons treated with astrocyte-conditioned medium (ACM). We also examined the lifespan, performed climbing assays and analysed immunohistochemical data in pan-glial TDP-43-expressing flies in the presence or absence of a *Ptp61f* RNAi transgene.

**Results:**

PTP1B inhibition suppressed TDP-43-induced secretion of inflammatory cytokines (interleukin 1 beta (IL-1β), interleukin 6 (IL-6) and tumour necrosis factor alpha (TNF-α)) in primary astrocytes. Using a neuron-astrocyte coculture system and astrocyte-conditioned media treatment, we demonstrated that PTP1B inhibition attenuated neuronal death and mitochondrial dysfunction caused by overexpression of TDP-43 in astrocytes. In addition, neuromuscular junction (NMJ) defects, a shortened lifespan, inflammation and climbing defects caused by pan-glial overexpression of TDP-43 were significantly rescued by downregulation of *ptp61f* (the *Drosophila* homologue of PTP1B) in flies.

**Conclusions:**

These results indicate that PTP1B inhibition mitigates the neuronal toxicity caused by TDP-43-induced inflammation in mammalian astrocytes and *Drosophila* glial cells.

## Background

Transactive response DNA binding protein of 43 kDa (TDP-43) is a major component of cytoplasmic aggregates in neurons and glia in most patients with amyotrophic lateral sclerosis (ALS) and in a subgroup of patients with frontotemporal lobar degeneration with TDP-43 (FTLD-TDP) [1–3]. TDP-43 is a type of heterogeneous nuclear ribonucleoprotein (hnRNP) that is ubiquitously expressed and concentrated in the nucleus [4]. More than 50 missense mutations in *TARDBP* have been identified in sporadic and familial cases of ALS [5]. Many transgenic animal models expressing wild-type (WT) or mutant TDP-43 have been generated, most of which mimic key clinical features found in ALS patients, such as impaired motor function, neurodegeneration and accumulation of cytoplasmic TDP-43 aggregates [6–10].

Cytoplasmic aggregation of TDP-43 is one of the major features in TDP-43 proteinopathy [1, 11, 12], and these aggregates are associated with many neurodegenerative diseases, including FTLD, ALS and Alzheimer’s disease (AD) [3, 13, 14]. This cytoplasmic accumulation of TDP-43 eventually leads to neuronal toxicity. Several lines of evidence indicate that TDP-43 is ubiquitously expressed in many tissues and cell types, including glial cells of the central nervous system. The inflammatory activation of astrocytes and/or microglia is prevalent in most animal models of TDP-43 proteinopathy, such as disease-associated transgenic mice with mutations in TDP-43 and SOD1 [15]. Moreover, several studies have shown that the expression of mutant SOD1 in astrocytes and microglia significantly exacerbates neurodegeneration [16–20]. In particular, selective expression of TDP-43 in rat astrocytes also leads to non-cell autonomous neuronal toxicity [21]. These data suggest that nonneuronal cells, such as microglia and astrocytes, contribute to neuronal toxicity in TDP-43 proteinopathy.

Protein tyrosine phosphatase 1B (PTP1B) regulates many important signalling pathways that are relevant to ALS, such as inflammation and ER stress. Previous studies suggest that inhibition of PTP1B is associated with early signalling in macrophages in response to inflammation [22]. Moreover, IL-4-induced anti-inflammatory features are negatively regulated by PTP1B [23]. PTP1B is also associated with microglia-mediated neuroinflammation. Recent studies suggest that high-fat diet-induced activation of hypothalamic microglia is significantly attenuated by PTP1B deficiency [24]. LPS-induced neuroinflammation in microglia is also mitigated by PTP1B inhibition [25]. In addition, a recent study indicated that ER stress-induced neuronal toxicity is dramatically reduced by PTP1B inhibition in *Drosophila* and mammalian neurons [26]. However, it has never been determined whether PTP1B is implicated in the proinflammatory activation of astrocytes.

In the present study, we found that PTP1B expression in astrocytes was upregulated by TDP-43 overexpression and that PTP1B inhibition attenuated the TDP-43-induced proinflammatory response of astrocytes. By utilizing a pan-glial TDP-43 proteinopathy Drosophila model and mouse primary cell culture model, we showed that PTP1B is a critical mediator of the neuronal toxicity caused by TDP-43-induced neuroinflammation. Therefore, targeting PTP1B may represent a promising therapeutic intervention for neurodegenerative diseases with TDP-43 proteinopathy.

## Methods

### Reagents

The following reagents were purchased as indicated: dimethyl sulfoxide (DMSO) and all-*trans* retinoic acid (RA) [Sigma]; PTP1B inhibitor [Calbiochem/Merck-Millipore]; and recombinant mouse IL-1β protein, recombinant mouse IL-6 protein, and recombinant mouse TNF-α protein [R&D Systems].

### Antibodies

The following antibodies were used for immunoblotting: mouse anti-TurboGFP (TA150041) [Origene]; rabbit anti-TDP-43 (10782-2-AP) [Proteintech]; mouse anti-Lamin A/C (05-714) [EMD Millipore]; rabbit anti-beta Actin (ab16039) [Abcam]; rabbit anti-NF-κB p65 (Ser536) (3033), rabbit anti-NF-κB (8242) and HRP-conjugated anti-α-tubulin (9099) [Cell Signaling Technology]; and rabbit anti-PTP1B (sc-14021), HRP-conjugated anti-rabbit IgG (sc-2004) and HRP-conjugated anti-mouse IgG (sc-2005) [Santa Cruz]. The following antibodies were used for immunocytochemistry (ICC): rabbit anti-MAP2 (1:500; AB5622) and TRITC-conjugated phalloidin (1:500; 90228) [Millipore]. The following antibody was used for immunohistochemistry (IHC): FITC-conjugated anti-HRP (1:150; 123-095-021) [Jackson ImmunoResearch Laboratories]. The following antibodies were used for neutralizing target proteins: rabbit anti-IL-1β (ab9722) [Abcam]; rat anti-TNF-α (14-7321-81) [Invitrogen]; rabbit anti-IgG (2729) and mouse anti-IgG (5415) [Cell Signaling Technology]; and mouse anti-IL-6 (sc-57315) and rat anti-IgG (sc-2026) [Santa Cruz].

### Primary cell cultures

Primary cultures of dissociated cerebral cortical neurons were prepared from C57/BL6 16-day-old embryonic mice as described previously [27, 28]. Briefly, mouse embryos were decapitated, and the brains were rapidly removed and placed in a culture dish containing HBSS (Gibco). Cortices were isolated, transferred to a conical tube and washed twice in HBSS (Gibco). Cortical tissues were enzymatically digested with prewarmed papain (20 units/ml) (Worthington Biochemical Corporation) and DNase I (0.005%) for 30 min at 37 °C. The tissues were mechanically dissociated (triturated) with 1000 μl and 200 μl pipette tips to obtain complete tissue homogenization. Cortical cells were centrifuged at 130×*g* for 10 min at room temperature, and the dissociated cells that were obtained were seeded onto plates coated with poly-d-lysine (Sigma-Aldrich) in neurobasal media containing 2 mM glutamine (Gibco), N2 supplement (Gibco), B27 supplement (Gibco) and 50 μg/ml penicillin-streptomycin (P/S, Gibco). Neuronal purity was determined by calculating the ratio of MAP2-positive cells to total viable cells (Figure S1a; upper). The culture media were changed initially after 5 days and every 3 days thereafter, and cells were used after being cultured for 14–21 days.

Primary astrocyte cultures were prepared from 1- to 2-day-old C57/BL6 mice as described previously [29]. Briefly, whole brains were homogenized and passed through a 70-μm strainer. Cells were seeded in T75 culture flasks. Cells were grown at 37 °C in a humidified atmosphere containing 5% CO_2_. Culture medium was changed initially after 5 days and every 2 days thereafter, and cells were used after being cultured for 14–21 days. Secondary pure astrocyte cultures were obtained by shaking mixed glial cultures at 250 rpm for 4 h; then, the culture medium was discarded. Astrocytes were dissociated using trypsin-EDTA (Life Technologies) and then were centrifuged at 800×*g* for 30 min. The astrocytes obtained were seeded onto plates in DMEM (Life Technologies) supplemented with 10% heat-inactivated foetal bovine serum (FBS; Gibco) and 50 μg/ml P/S. The purity of the cells in culture was determined by immunocytochemistry, which found that the cultures contained over 93% GFAP-positive cells (Figure S1a; lower). Animals used in the current research were acquired and cared for in accordance with the guidelines published in the National Institutes of Health *Guide for the Care and Use of Laboratory Animals*.

### Transfection

Primary astrocytes in 6-well plates (40 × 10^4^ cells/well) were transfected with 4 μg of *Gfp* (*pCMV6-AC-Gfp*, Origene Technologies, PS100010) or human *TDP*-*43* (*pCMV6*-*AC*-*TDP*-*43*-*Gfp*, Origene Technologies, RG210639) vectors using Lipofectamine 3000 reagent (Invitrogen). At 2 days posttransfection, cells were subjected to FACS of *Gfp*-transfected live cells. The *Gfp*-transfected sorted cells (8 × 10^4^ cells/well) were treated with a PTP1B inhibitor (PTP1Bi, 5 μM) or DMSO for 24 h. A total experimental duration of 3 days posttransfection was chosen to reduce the transfection-associated background toxicity.

### siRNA transfection

Primary astrocytes in 6-well plates (40 × 10^4^ cells/well) were cotransfected with a *pCMV6*-*AC*-*TDP*-*43*-*Gfp* vector and control siRNA (Dharmacon; D-001810-10) or mouse *Ptb1b* siRNA (Dharmacon; L-040818-00) using Lipofectamine 3000 reagent (Invitrogen) or Lipofectamine RNAiMAX reagent (Invitrogen), and then the cells were incubated for 3 days. The downregulation of target protein expression in the transfected cells was confirmed by immunoblot analysis. At 72 h posttransfection, cells were subjected to FACS of *Gfp*-transfected live cells, and the sorted cells were then fixed or harvested for further analyses.

### Cytotoxicity tests

Primary astrocytes (5 × 10^4^ cells/well) were grown in 96-well plates and treated with a PTP1B inhibitor (PTP1Bi, 5 μM) for 24 h. Cells cultured in an equal volume of DMSO were used as a control. To measure cytotoxicity, Cell Counting Kit-8 (CCK-8; Enzo Life Science) was used in accordance with the manufacturer’s instructions. Briefly, 10 μl of CCK-8 reagent was added to each well, and the plate was incubated at 37 °C for 2 h. Absorbance was measured at 450 nm using a microplate reader (Tecan). Cell viability was expressed as a percentage of control (DMSO-treated) cell viability. All experiments were performed in triplicate.

### Quantitative RT-PCR

RNA was extracted from cells and fly heads using TRIzol reagent (Life Technologies). RNA cleanup was performed using an RNeasy Mini Kit (QIAGEN) according to the manufacturer’s instructions. cDNA synthesis was performed at 37 °C for 120 min with 100 ng of RNA using a High Capacity cDNA Reverse Transcription Kit (Applied Biosystems). Quantitative RT-PCR was performed using a One-Step SYBR® PrimeScript™ RT-PCR Kit (Perfect Real Time; Takara Bio Inc.) according to the manufacturer’s instructions, which was followed by detection using an Applied Biosystems 7500 Real-Time PCR system (Applied Biosystems). *Gapdh* and *18S rRNA* were used as internal controls. The 2^−ΔΔCt^ method was used to calculate relative differences in gene expression that were determined by real-time PCR experiments [30].

### Immunoblot analysis

For total protein extraction, either cells or 20 adult fly heads were homogenized in RIPA buffer (Cell Signaling Technology) or 1× LDS sample buffer (Invitrogen) containing a protease and phosphatase inhibitor cocktail (Roche). The protein concentration of the cell lysates was determined using a BCA protein assay kit (Thermo Fisher Scientific). Next, the protein extracts were mixed with 4× NuPAGE LDS sample buffer (Invitrogen) and 10× NuPAGE Sample Reducing Agent buffer (Invitrogen), and then they were boiled at 95 °C for 5 min. An equal amount of protein from each sample was separated on NuPAGE 4-12% Bis-Tris gels (Novex) or NuPAGE 3-8% Tris-acetate gels (Novex) and then was transferred to a polyvinylidene difluoride (PVDF; Novex) membranes using a transfer apparatus according to the manufacturer’s protocol (Novex). The membranes were blocked with 5% skim milk and were sequentially incubated with primary antibodies and HRP-conjugated secondary antibodies (anti-rabbit IgG and anti-mouse IgG), followed by detection with an ECL Prime kit (Amersham Biosciences). Samples from three independent experiments were used in this analysis. The relative expression level was determined using Fusion-FX software (Vilber Lourmat).

### Nuclear and cytoplasmic fraction extraction

*TDP*-*43*-transfected astrocytes (15 × 10^4^ cells/well) were fractionated using NE-PER nuclear and cytosolic extraction reagents (Thermo Fisher Scientific). Nuclear and cytoplasmic fractions in ice-cold CER I and CER II buffer were obtained by centrifugation at 16,000 × g for 5 min at 4 °C. Supernatants containing the cytoplasmic extract were harvested, and the pellets were solubilized in ice-cold NER buffer. After vortexing, the extracts were centrifuged at 16,000 × g for 10 min at 4 °C. Supernatants containing the nuclear extract were harvested. The extracts were mixed with 4× NuPAGE LDS sample buffer and 10× NuPAGE Sample Reducing Agent buffer and then were boiled at 95 °C for 5 min.

### Elisa

To determine IL-1β, IL-6 and TNF-α protein levels, supernatants of *TDP*-*43*-transfected astrocytes (15 × 10^4^ cells/well) were analysed using mouse ELISA Development Kits for each cytokine or chemokine (R&D Systems). Briefly, 96-well ELISA plates were coated with the capture antibodies. After blocking those antibodies, samples or recombinant cytokine or chemokine standards were added. For detection, biotinylated detection antibodies were added, which was followed by incubation with streptavidin-HRP and substrate (R&D Systems) according to the manufacturer’s instructions. The absorbance was measured at 450 nm and 540 nm using a microplate reader (Tecan).

### Astrocyte-conditioned media

To produce PTP1B inhibitor-treated astrocyte-conditioned medium (ACM), *Gfp*/*TDP*-*43*-transfected live primary astrocytes (15 × 10^4^ cells/well) that were obtained via FACS were allowed to acclimate for 24 h in DMEM. *Gfp*- or *TDP*-*43*-transfected primary astrocytes were treated with a PTP1B inhibitor (PTP1Bi, 5 μM) or DMSO for 24 h. Primary astrocytes were then washed twice with PBS and cultured in fresh DMEM for an additional 24 h. The ACM was then collected, centrifuged at 200×*g* for 10 min to remove cell debris and stored at − 80 °C until further analysis.

To generate *Ptp1b* siRNA-transfected ACM, live primary astrocytes (15 × 10^4^ cells/well) that were cotransfected with a *Gfp*/*TDP*-*43* expression construct and a *Ptp1b* siRNA or control siRNA were selected via FACS were allowed to acclimate for 24 h in DMEM. Primary astrocytes were then washed twice with PBS and cultured in fresh DMEM for an additional 24 h. The ACM was then collected, centrifuged at 200×*g* for 10 min to remove cell debris and stored at − 80 °C until further analysis. To obtain the control ACM, cells were cultured in DMEM supplemented with 10% FBS and 50 μg/ml P/S.

To generate IL-1β, IL-6 or TNF-α antibody-neutralized ACM, *Gfp*/*TDP*-*43*-transfected live primary astrocytes (15 × 10^4^ cells/well) obtained via FACS were allowed to acclimate for 24 h in DMEM. *Gfp*- or *TDP*-*43*-transfected primary astrocytes were treated with an IL-1β antibody (50 ng/ml), an IL-6 antibody (50 ng/ml), a TNF-α antibody (100 ng/ml) and a control IgG (100 ng/ml) for 1 h. Primary astrocytes were then washed twice with PBS and cultured in fresh DMEM for an additional 24 h. The ACM was then collected, centrifuged at 200×*g* for 10 min to remove cell debris and stored at − 80 °C until further analysis.

### ACM-treated neuron culture

For ACM-treated neuron culture, primary cortical neurons were stimulated with *Gfp*-transfected + DMSO-treated ACM, *Gfp*-transfected + PTP1B inhibitor-treated ACM, *TDP*-*43*-transfected + DMSO-treated ACM and *TDP*-*43*-transfected + PTP1B inhibitor-treated ACM (GFP ACM, GFP + PTP1B ACM, TDP-43 ACM and TDP-43 + PTP1Bi ACM, respectively) for 24 h, and then the cells were subjected to a CCK-8 assay. Primary cortical neurons were also treated with *Gfp* + control siRNA, *Gfp* + *Ptp1b* siRNA, *TDP*-*43* + control siRNA and *TDP*-*43* + *Ptp1b* siRNA cotransfected ACM (GFP ACM, GFP + *Ptp1b* siRNA ACM, TDP-43 ACM and TDP-43 + *Ptp1b* siRNA ACM, respectively) for 5 days, and then the cells were subjected to a CCK-8 assay.

### Neuron-astrocyte coculture

For neuron-astrocyte coculture, *Gfp*/*TDP*-*43*-transfected live primary astrocytes (8 × 10^4^ cells/well) that were selected via FACS were allowed to acclimate for 24 h in fresh DMEM. *Gfp*/*TDP*-*43*-transfected primary astrocytes were treated with a PTP1B inhibitor (PTP1Bi, 5 μM) or DMSO for 24 h. Primary astrocytes were then washed twice with PBS, detached, and seeded in the upper compartment of transwell inserts (8 μm pore membrane; Millipore) at a density of 4 × 10^4^ cells/well. Primary cortical neurons were plated in the lower compartment of 24-transwell plates at a density of 4 × 10^4^ cells/well. The primary cells were incubated at 37 °C for either 36 or 48 h and then were subjected to a CCK-8 assay.

### CMFDA staining analysis

Chloromethylfluorescein diacetate (CMFDA) is a long-term cell-tracing green dye that efficiently stains all cells [31, 32]. A CMFDA fluorescence-based assay was used to quantify the relative changes in neuronal cell viability. Primary cortical neurons were labelled with a 5 μM concentration of CellTracker Green CMFDA Dye (Invitrogen) for 30 min according to the manufacturer’s protocol. Then, the dye solution was aspirated, and the cells were incubated with dye-free medium for 45 min. The samples were mounted and observed with a microscope. Photomicrographs from three randomly chosen fields were obtained, and the number of CMFDA-positive cells was counted. The quantification of CMFDA-positive cells is represented as a percentage of the control.

### NSC-34 cell culture

NSC-34 cells (cat. no. CLU140; Cedarlane) are well-characterized lower motor neuron-like cells generated by the fusion of embryonic mouse spinal cord cells and mouse neuroblastoma cells [33]. NSC-34 cells show morphological and physiological similarities to mature primary motor neurons. NSC-34 cells were maintained in DMEM supplemented with 10% FBS, 50 μg/ml P/S and 2 mM glutamine. The differentiation of NSC-34 cells was performed as described previously [34, 35]. For differentiation, NSC-34 cells were grown to confluence, and the growth medium (DMEM + 10% FBS) was exchanged for differentiation medium (1:1 DMEM/Ham’s F12 supplemented with 1% FBS, 1% MEM-NEAA, 50 μg/ml P/S and 1 μM all-*trans* retinoic acid (RA)) every 2 days.

### Immunocytochemistry analysis

Cells were fixed in 4% or 8% paraformaldehyde in PBS (Gibco, 70011-044) for 30 min at room temperature. The cells were then washed three times with PBS and permeabilized in PBS-T (0.3% Triton X-100) for 15 min at room temperature. After blocking with 10% normal goat serum in PBS-T for 1 h, primary antibodies and 2% normal goat serum in PBS-T were incubated with the cells overnight at 4 °C. The cells were then washed three times with PBS-T and incubated with an Alexa-conjugated secondary antibody for 1 h at room temperature. Alexa 594-conjugated goat anti-rabbit IgG (1:500; Jackson ImmunoResearch Laboratories, 111-585-144), Alexa 488-conjugated anti-rabbit IgG antibody (1:500; Jackson ImmunoResearch Laboratories, 111-545-144), Alexa 594-conjugated anti-mouse IgG antibody (1:500; Jackson ImmunoResearch Laboratories, 111-585-146) and Alexa 647-conjugated anti-goat IgG antibody (1:500; Jackson ImmunoResearch Laboratories, 705-605-147) were used as secondary antibodies as indicated. Then, samples were mounted and observed with a fluorescence microscope (Nikon).

### Mitochondrial activity assay

For assessment of neuronal mitochondrial dysfunction, primary cortical neurons that were grown in XF24-well culture plates (Seahorse Bioscience) were stimulated with GFP ACM, TDP-43 ACM or TDP-43/PTP1B inhibitor-treated ACM for 5 days. After the treatments, the cells were washed twice with XF Base Medium supplemented with 2 mM l-glutamine, 10 mM d-glucose and 1 mM sodium pyruvate (pH 7.4) and incubated at 37 °C in a non-CO_2_ incubator for 1 h. Mitochondrial dysfunction was evaluated using an XF Cell Mito Stress Test Kit (Seahorse Bioscience) according to the manufacturer’s instructions, followed by measurement using an XF24 Extracellular Flux Analyzer (Seahorse Bioscience). The 24-well utility plate was hydrated, treated with 2 μM oligomycin, 1 μM carbonyl cyanide 4-(trifluoromethoxy) phenylhydrazone (FCCP), 0.5 μM antimycin A+rotenone, and then calibrated by the analyser. The basal oxygen consumption rate (OCR), ATP production, maximum reserve and respiratory capacity were calculated as previously described [36], and averages were calculated from five wells per condition in each individual experiment. The OCR was normalized to the total protein concentration (OD). After the seahorse analysis, the plate was centrifuged at 280×*g* for 5 min. The media were aspirated and washed twice with PBS. Cells were lysed in RIPA buffer. Protein concentrations of cell lysates were determined using a BCA assay kit.

### Quantification of dendritic spines

Cells were fixed in 8% paraformaldehyde in PBS (Gibco) for 30 min at room temperature. The cells were then washed three times with PBS and permeabilized in PBS-T (0.3% Triton X-100) for 15 min at room temperature. After blocking with 10% normal goat serum in PBS-T for 1 h, a MAP2 antibody with 2% normal goat serum in PBS-T was incubated with the cells overnight at 4 °C. The cells were then washed three times with PBS-T and incubated with TRITC-conjugated phalloidin and an Alexa 594-conjugated goat anti-rabbit IgG (1:500) for 1 h at room temperature. Then, samples were mounted and observed with a confocal microscope (Nikon). Fifteen dendrites were randomly selected from four separate cortical neuron cultures in the GFP ACM, TDP-43 ACM and TDP-43 + PTP1Bi ACM-treated cortical neuron groups. Images of dendritic spine were taken from 7 raw images of 4 independent primary cortical neuron cultures per ACM-treated group, and 1 or 2 images were excluded from each group because the spine analysis was disturbed by crossing dendrite images. Each image was acquired to z-stack image series, which included 15 frames with 200 nm sections using a × 100 objective (FOV 1024 BY 1024) with a Nikon A1Rsi confocal microscope. The z-stack image that was selected contained a dendritic segment approximately 50 μm in length that was distal to a dendritic branch point, and it was manually analysed for additional dendritic branch points. Dendritic spines were analysed for dendritic protrusions that could not be classified as stubby, mushroom, thin spine or filopodia. The maximum spine length and minimum spine end diameter were set at 5 μm and 0.2 μm, respectively. The dendritic spine morphological changes were analysed using Imaris software (Imaris, Bitplane, Inc.), and data were exported into Excel (Microsoft). Statistical analysis was then conducted using Prism 8 (GraphPad Prism Software, La Jolla, CA). All values are presented as the mean ± SEM, with *N* indicating the number of replicates. The corresponding *p* values are described in the figure legend of each figure.

### Fly strains

*Drosophila* stocks were maintained on standard cornmeal agar media at 24 °C unless otherwise noted. The construct UAS-TDP-43 was described previously [11]. *Repo*-*Gal4*/*+* and *Repo*-*Gal4*/*UAS*-*TDP*-*43* have previously been described [37]. The upstream activation sequence-RNA interference (UAS-RNAi) line against *Ptp61f* or *Egfp* was obtained from the Bloomington stock centre (Bloomington, IN; http://flystocks.bioindiana.edu/). We crossed females from UAS-RNAi lines (*Egfp RNAi*^*VALIUM20-EGFP.shRNA.4*^and *Ptp61f RNAi* ^*HMS004*^) with males harbouring the Repo-Gal4 (pan-glial) driver to knock down target genes in the entire glial cell population.

### Immunohistochemical analysis

Wandering third instar larvae of adult flies were randomly selected, dissected in PBS and then fixed in 4% formaldehyde in PBS for approximately 15 min. After blocking with 5% normal goat serum in PBS-T (0.3% Triton X-100) for 1 h, the antibodies with 5% normal goat serum in PBS-T were incubated with the fixed larvae for approximately 1.5 h at room temperature. Larval preparations were mounted with a SlowFade Antifade Kit (Invitrogen). NMJ images were visualized using a laser scanning confocal microscope system (TCS SP5 AOBS/Tandem microscope, Leica-Microscope Systems GmbH, Germany) at Korea Basic Science Institute, Gwangju Center. Leica Application Suite Advanced Fluorescence software was used to analyse images. We performed the analyses of NMJs essentially as described [38].

### Lifespan and adult climbing assays

We performed lifespan and climbing assay using offspring from the crosses of *Egfp RNAi*^*VALIUM20-EGFP.shRNA.4*^ or *Ptp61f RNAi* ^*HMS0042*^ with Repo-Gal4 lines. Adult males (0 to 1 day old) were separated and transferred into experimental vials at a density of 20 (for lifespan) or 25 (for climbing assay) flies per vial (*n* > 100). The number of dead flies was scored daily, and flies were transferred to fresh media or paper every other day. Adult locomotor function was assessed by a previously described method [39], and there were 125 flies per genotype per time point in all experiments. Experiments were repeated twice to ensure consistent results.

### Statistical analyses

Data were analysed by Student’s *t* test (Vassar Stats, www.vassarstats.net) or were first analysed using one-way ANOVA followed by Bonferroni’s multiple comparison test (GraphPad Prism Software, La Jolla, CA). Differences were considered significant when *p* < 0.05 and are indicated as follows: *p < 0.05; **p < 0.005; ***p < 0.001; or N.S., not significant.

## Results

### PTP1B is an essential modulator of TDP-43-induced inflammation in astrocytes

**Fig. 1:**
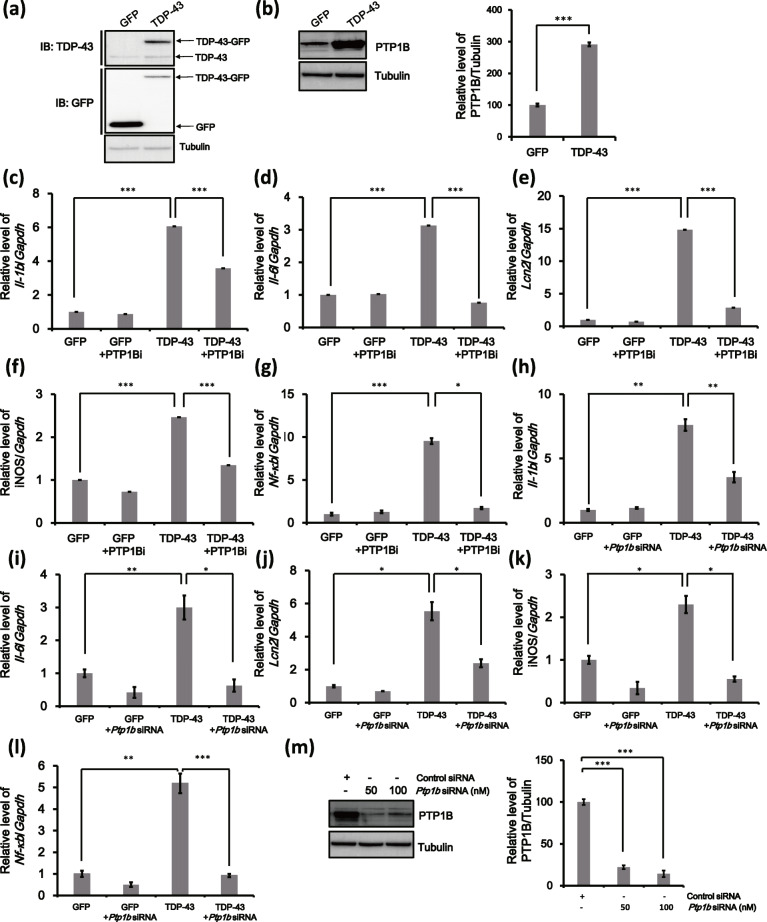
Expression levels of PTP1B and proinflammatory genes are upregulated primary astrocytes by TDP-43 overexpression in **a**, **b**, astrocytes were transfected with a GFP or TDP-43-GFP expression construct, and after 3 days, FACS of *Gfp*-transfected live cells was performed. **a** Successful transfection of *Gfp*- or *TDP-43-Gfp* in cells was confirmed by immunoblotting using an anti-TDP-43 or anti-GFP antibody. Tubulin was used for normalization. **b** Immunoblot analysis of *Gfp*- or *TDP-43-Gfp*-transfected cells was performed to detect the expression of PTP1B proteins. Tubulin was used for normalization. Data are presented as the mean ± SD. ***p < 0.001 (Student’s *t* test). **c**–**g** *TDP-43-Gfp*-transfected astrocytes were treated with a PTP1B inhibitor (PTP1Bi, 5 μM) for 1 day, and then real-time PCR was performed. mRNA transcript levels are presented as the mean ± SD. *Gapdh* was used for normalization. *p < 0.05; ***p < 0.001 (one-way ANOVA). **h**–**l** Astrocytes were cotransfected with a *TDP-43* expression construct and either a control siRNA or a mouse *Ptp1b* siRNA for 3 days; then, FACS of *Gfp*-transfected live cells was performed. These cells were allowed to acclimate for 1 day and then were subjected to real-time PCR experiments. mRNA transcript levels are presented as the mean ± SD. *Gapdh* was used for normalization. *p < 0.05; **p < 0.005; ***p < 0.001 (one-way ANOVA). **m** Astrocytes were transfected with a *Ptp1b*-specific siRNA (50 or 100 nM) for 48 h. Immunoblot analysis for the expression level of PTP1B protein. Tubulin was used for normalization. Data are presented as the mean ± SD. ***p < 0.001 (one-way ANOVA)

To investigate the interaction between TDP-43 and PTP1B in astrocytes, we first sorted GFP-positive cells among *Gfp*- and GFP-tagged *TDP*-*43*-transfected astrocytes and then assessed the levels of PTP1B by immunoblotting. PTP1B expression was significantly increased after TDP-43 transfection in primary astrocytes compared to *Gfp*-transfected cells (Fig. 1a, b). Furthermore, TDP-43 protein levels were also greatly increased in the insoluble fraction of TDP-43-overexpressing cells (Figure S2a). Lipofectamine-only treatment did not affect the viability of primary astrocytes (Figure S3a). Recent data have shown that PTP1B is a positive regulator of neuroinflammation in microglia [25]. In line with this evidence, we also observed that TDP-43 overexpression in primary astrocytes upregulated the expression of inflammatory genes (*Il*-*1b*, *Il*-*6*, *Lcn2*, *iNOS*, or *Nf*-*κb*) (Fig. 1c–l) compared to the levels observed after GFP overexpression in primary astrocytes, further supporting the idea that TDP-43 overexpression induced inflammation in primary astrocytes. To rule out the possibility of off-target effects resulting from the PTP1B inhibitor, we treated cells with an siRNA against the *Ptp1b* gene. The protein level of PTP1B was markedly decreased by *Ptp1b* siRNA transfection in primary astrocytes (Fig. 1m). Real-time PCR analysis using *Gapdh* or *18S rRNA* as an internal control indicated that the TDP-43-induced inflammatory gene level was similarly decreased by PTP1B inhibitor treatment and by *Ptp1b* siRNA transfection of mouse primary astrocytes (Figs. 1c–l and S4a-j). Taken together, our data demonstrated that TDP-43 overexpression activates the inflammatory response and that PTP1B is a critical regulator of TDP-43-induced neuroinflammation in primary astrocytes.

### PTP1B regulates TDP-43-induced inflammation via the NF-κB pathway

**Fig. 2:**
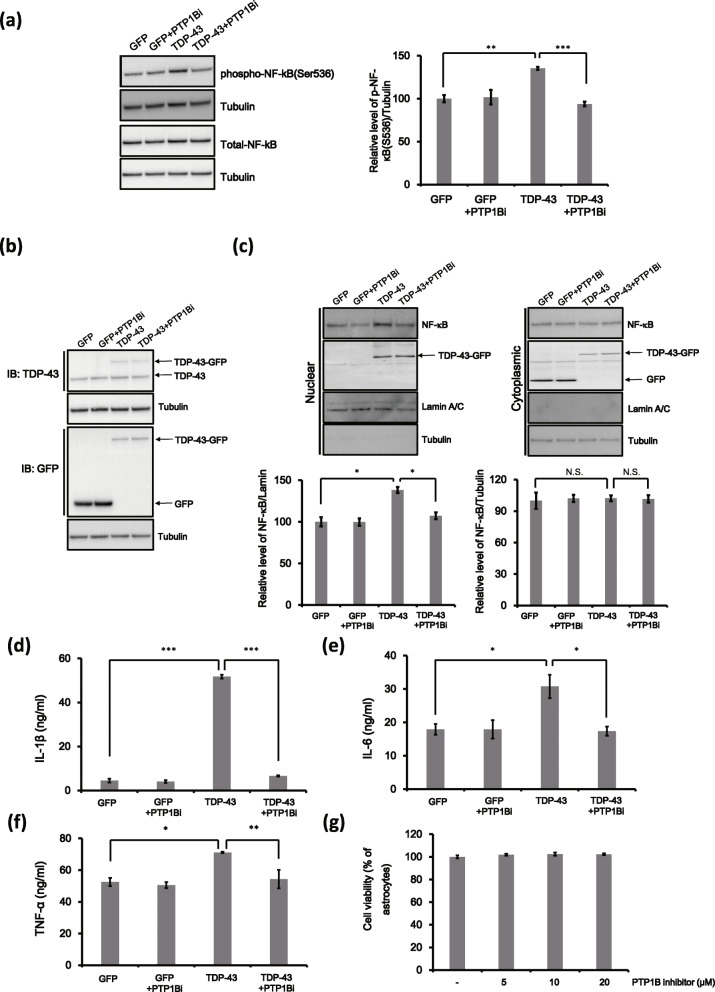
PTP1B inhibition suppresses TDP-43-induced inflammation via the NF-κB pathway. **a**–**c** *TDP-43-Gfp*-transfected astrocytes were treated with PTP1B inhibitor (PTP1Bi, 5 μM) for 24 h and then were analysed by immunoblotting. **a** Immunoblot analysis of *Gfp-* or *TDP-43-Gfp*-transfected cells was performed to detect the protein expression of phosphorylated NF-κB (Ser536) and total NF-κB after PTP1B inhibitor (PTP1Bi, 5 μM) treatment for 24 h. Tubulin was used for normalization. Data are presented as the mean ± SD. **p < 0.005; ***p < 0.001 (one-way ANOVA). **b** Expression of *Gfp-* or *TDP-43-Gfp* in cells was confirmed by immunoblotting using an anti-TDP-43 or anti-GFP antibody. Tubulin was used for normalization. **c** Cells were fractionated into nuclear and cytoplasmic extracts. Immunoblot analysis was performed for NF-κB protein in the nuclear and cytoplasmic fractions. Lamin A/C (nuclear fraction) and tubulin (cytoplasmic fraction) were used for normalization. Data are presented as the mean ± SD. *p < 0.05; *N.S.* not significant (one-way ANOVA). **d**–**f** *Gfp-* or *TDP-43-Gfp*-transfected astrocytes were treated with a PTP1B inhibitor (PTP1Bi, 5 μM) for 24 h, and then the ACMs were harvested. ELISAs were conducted for secreted cytokines (IL-1β, IL-6 and TNF-α) in the GFP ACM, TDP-43 ACM and TDP-43 + PTP1Bi ACM groups. Data are presented as the mean ± SD. *p < 0.05; **p < 0.005; and ***p < 0.001 (one-way ANOVA). **g** A CCK-8 assays were performed to assess the viability of primary astrocytes treated with the PTP1B inhibitor (PTP1Bi, 5 μM, 10 μM or 20 μM) for 24 h. Data are presented as the mean ± SD

Next, we investigated the mechanism by which PTP1B regulates the TDP-43-induced inflammatory response. A previous study showed that TNF-α-induced PTP1B upregulation results in the activation of the NF-κB pathway in rat hypothalamic organotypic cultures [40]. Furthermore, TDP-43 protein treatment activates microglia via the NF-κB pathway [41]. Thus, we postulated that the NF-κB pathway is also involved in TDP-43-mediated neuroinflammation in astrocytes. To support this hypothesis, we extracted proteins from *Gfp*- and *TDP*-*43*-transfected astrocytes and then assessed the phosphorylation level of NF-κB subunit p65 using a phospho-specific (Ser536) anti-p65 antibody. Ser536 phosphorylation of p65 is required for nuclear translocation of NF-κB, and nuclear translocation of the NF-κB complex induces expression of inflammatory genes [42]. NF-κB p65 (Ser536) phosphorylation was significantly increased in *TDP*-*43*-transfected cells compared to *Gfp*-transfected cells (Fig. 2a). Furthermore, TDP-43-induced NF-κB p65 phosphorylation (Ser536) was restored by PTP1B inhibition (Fig. 2a; 135 ± 1.7% in the TDP-43 group, and 93 ± 2.6% in the TDP-43 + PTP1Bi group; all test groups were compared to the *Gfp* transfected astrocyte group). PTP1B inhibition did not affect total NF-κB protein levels in *Gfp*- or *TDP*-*43*-transfected cells (Fig. 2a).

Next, we performed immunoblotting to determine the effect of PTP1B inhibition on TDP-43 protein levels. The results showed that the increased TDP-43 protein level in TDP-43-overexpressing cells was not changed by PTP1B inhibition; thus, PTP1B does not seem to directly affect TDP-43 protein levels (Fig. 2b).

Nuclear-cytoplasmic fractionation of primary astrocytes using NE-PER Nuclear and Cytoplasmic Extraction Reagents was employed to determine whether PTP1B regulates the nuclear translocation of p65 in response to TDP-43. The level of nuclear NF-κB p65 was significantly higher in *TDP*-*43*-transfected astrocytes than it was in *Gfp*-transfected cells, and PTP1B inhibition effectively suppressed the TDP-43-induced nuclear translocation of NF-κB p65 (Fig. 2c 21.5 ± 1.3% in the TDP-43 group versus 9.3 ± 1.4% in the TDP-43 + PTP1Bi group; all test groups were compared to the *Gfp*-transfected astrocyte group).

We also assessed the levels of inflammatory cytokines and chemokines using a cytokine proteome profiler array (blotting) in the ACM obtained from GFP-expressing astrocytes, TDP-43-expressing astrocytes and TDP-43-GFP + PTP1Bi-treated astrocytes (data not shown). Notably, we found that the secretion levels of interleukin 1 beta (IL-1β), interleukin 6 (IL-6) and tumour necrosis factor alpha (TNF-α) were greatly increased in the TDP-43 ACM group compared with the GFP ACM group. Next, we measured the concentrations of IL-1β, IL-6 and TNF-α in the ACM using ELISA Development Kits (R&D Systems; Fig. 2d–f). These 3 proinflammatory cytokines were markedly upregulated in the ACM obtained from TDP-43-overexpressing astrocytes (Fig. 2d–f; IL-1β: 4.50 ± 0.8 ng/ml in the GFP ACM group versus 51.7 ± 1.5 ng/ml in the TDP-43 ACM group; IL-6: 17.9 ± 1.5 ng/ml in the GFP ACM group versus 30.7 ± 6.0 ng/ml in the TDP-43 ACM group; and TNF-α: 52.5 ± 1.4 ng/ml in the GFP ACM group versus 71.1 ± 0.2 ng/ml in the TDP-43 ACM group). Moreover, PTP1B inhibition suppressed the secretion of inflammatory cytokines caused by TDP-43 overexpression (Fig. 2d–f; IL-1β: 51.7 ± 1.5 ng/ml in the TDP-43 ACM group versus 6.6 ± 0.4 ng/ml in the TDP-43 + PTP1Bi ACM group; IL-6: 30.7 ± 6.0 ng/ml in the TDP-43 ACM group versus 17.3 ± 2.3 ng/ml in the TDP-43 + PTP1Bi ACM group; and TNF-α: 71.1 ± 0.2 ng/ml in the TDP-43 ACM group versus 54.3 ± 3.3 ng/ml in the TDP-43 + PTP1Bi ACM group). We also showed that the PTP1B inhibitor alone did not affect the viability of astrocytes (Fig. 2g). These data suggest that PTP1B inhibition mitigates TDP-43-induced inflammation via the NF-κB pathway in astrocytes.

### PTP1B inhibition and absorption of proinflammatory cytokines mitigate the neuronal toxicity caused by astrocytic TDP-43 overexpression

**Fig. 3:**
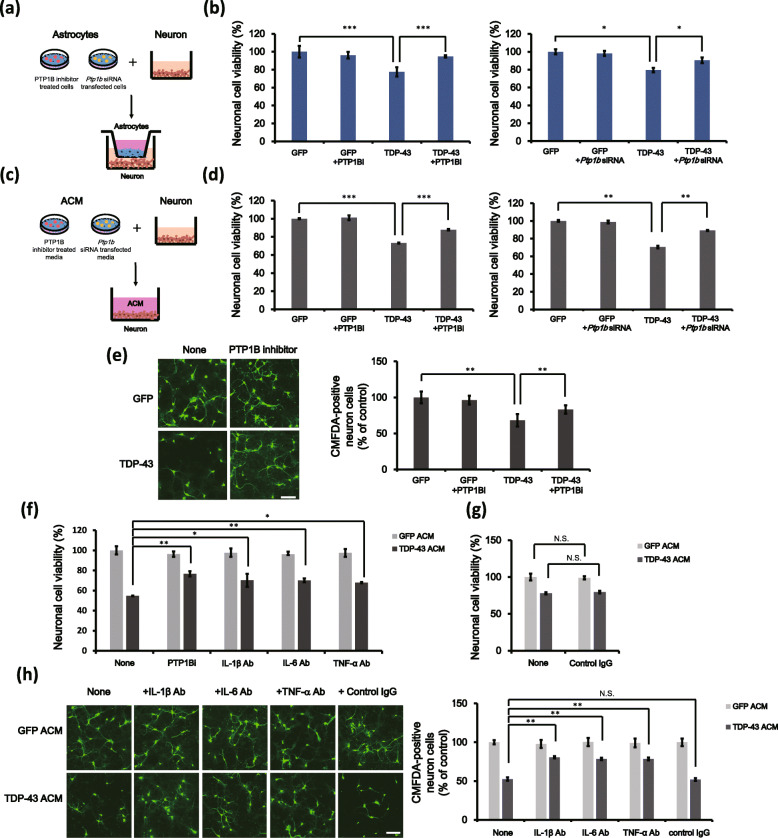
PTP1B inhibition and absorption of proinflammatory cytokines mitigate neuronal toxicity caused by TDP-43 overexpression in astrocytes. **a**, **b** Neuron-astrocyte coculture. **b** *TDP-43-Gfp*-transfected astrocytes were treated with a PTP1B inhibitor (PTP1Bi, 5 μM) or *Ptp1b* siRNA (50 nM) for 24 h and then were cocultured with primary cortical neurons in transwell culture inserts. Neuronal viability was measured using a CCK-8 assay after a coculture period of 36 or 48 h. Data are presented as the mean ± SD. *p < 0.05; ***p < 0.001 (one-way ANOVA). **c**–**e** ACM-treated neuron culture. **d** *TDP-43-Gfp*-transfected astrocytes were treated with a PTP1B inhibitor (PTP1Bi, 5 μM) or *Ptp1b* siRNA (50 nM) for 24 h, and then the ACM were harvested. Primary cortical neurons were stimulated with GFP ACM, TDP-43 ACM or TDP-43 + PTP1Bi ACM for 5 days, and then a CCK-8 assay was performed. Data are presented as the mean ± SD. **p < 0.005; ***p < 0.001 (one-way ANOVA). **e** At the end of neuron-ACM coculture, primary cortical neurons were stained with CMFDA (green). Then, CMFDA-positive neurons were counted under a fluorescence microscope. Data are presented as the mean ± SD of 3. **p < 0.005 (one-way ANOVA). Scale bars, 20 μm. **f**–**h** Primary cortical neurons stimulated with TDP-43 ACM were treated with IL-1β antibody (50 ng/ml), IL-6 antibody (50 ng/ml) and TNF-α antibody (10 ng/ml) for 5 days and then were subjected to a CCK-8 assay or to CMFDA staining. **f** CCK-8 assays were performed to assess the viability of primary cortical neurons. Data are presented as the mean ± SD. *p < 0.05; **p < 0.005 (one-way ANOVA). **g** CCK-8 assays were performed to assess the viability of primary cortical neurons stimulated with TDP-43-transfected ACM and treated with a control IgG antibody (50 ng/ml) for 5 days. Data are presented as the mean ± SD. *N*.*S*. not significant (Student’s *t* test). **h** CMFDA-positive neurons were counted under a fluorescence microscope. Data are presented as the mean ± SD. **p < 0.005; *N*.*S*. not significant (one-way ANOVA). Scale bars, 20 μm

To examine the effect of PTP1B on astrocytic TDP-43-induced neuronal toxicity, we used neuron-astrocyte transwell cocultures and ACM-treated neuron culture. Overexpression of TDP-43 in primary astrocytes caused neuronal toxicity in transwell cultures (Fig. 3a, b). This neuronal toxicity was significantly mitigated by PTP1B inhibitor treatment or *Ptp1b* siRNA transfection (Fig. 3b; 77 ± 5.1% in the TDP-43 group versus 94 ± 1.2% in the TDP-43 + PTP1Bi group, and all test groups were compared to the *Gfp*-transfected + DMSO-treated group; 79 ± 2.5% in the TDP-43-GFP + control siRNA group versus 90 ± 3.0% in the TDP-43-GFP + *Ptp1b* siRNA group, and all test groups were compared to the *Gfp* + control siRNA cotransfected group). We also showed that the PTP1B inhibitor alone did not affect neuronal viability. Similar to the results of the transwell culture assays, the ACM from TDP-43-overexpressing astrocytes with PTP1B inhibited showed lower neurotoxicity than the TDP-43 ACM in primary cortical neuron culture (Fig. 3d; 73 ± 0.5% in the TDP-43 ACM versus 87 ± 0.7% in the TDP-43-GFP + PTP1Bi ACM group, and all test groups were compared to *Gfp*-transfected + DMSO-treated ACM group; 82 ± 1.2% in the TDP-43-GFP + control siRNA ACM group versus 90 ± 0.3% in the TDP-43-GFP + *Ptp1b* siRNA ACM group, and all test groups were compared to *Gfp* + control siRNA cotransfected ACM group). We also confirmed the CCK-8 assay data by staining with the fluorescent cell tracker (green) CMFDA. The number of CMFDA-positive neurons was notably decreased in cocultures with *TDP*-*43*-*Gfp*-transfected astrocytes (68 ± 8.6%), and PTP1B downregulation significantly attenuated astrocytic TDP-43-induced neuronal death (Fig. 3e; 83 ± 5.8%). On the other hand, *Ptp1b* knockdown in astrocytes did not affect the viability of neuronal cells in the neuron-astrocyte transwell and ACM cocultures. Moreover, the ACM obtained from PTP1B inhibitor-treated astrocytes did not affect neuronal viability (Figure S5a).

We next investigated whether proinflammatory cytokines regulate neuronal toxicity under astrocytic TDP-43 overexpression. Our data showed that treatment with anti-IL-1β (50 ng/ml), anti-IL-6 (50 ng/ml) and anti-TNF-α (100 ng/ml) neutralizing antibodies suppressed TDP-43-induced neuronal toxicity. The concentration of the neutralizing antibodies used in the treatment was determined with reference to previous experiments [43–45]. The presence of an antibody targeting IL-1β, IL-6 or TNF-α dramatically attenuated neuronal toxicity treated with TDP-43 ACM in primary cortical neurons (Fig. 3f; IL-1β: 54.6 ± 0.3% in the TDP-43 ACM group versus 70.2 ± 6.4% in the TDP-43 ACM + anti-IL-1β antibody group; IL-6: 54.6 ± 0.3% in the TDP-43 ACM group versus 70.1 ± 1.8% in the TDP-43 ACM + anti-IL-6 antibody group; TNF-α: 54.6 ± 0.3% in the TDP-43 ACM group versus 67.8 ± 0.6% in the TDP-43 ACM + anti-TNF-α antibody group; all test groups were compared to the GFP ACM group). A control IgG antibody did not affect neuronal viability (Fig. 3g; 77.8 ± 1.3% in the TDP-43 ACM group versus 79.5 ± 1.8% in the TDP-43 ACM + control IgG antibody group; all test groups were compared to the GFP ACM group). To confirm these results, neurons were labelled with CMFDA dye, which functioned as a fluorescent cell tracker. Consistently, TDP-43 ACM treatment also decreased the number of CMFDA-positive cells, and treatment with a bioactive antibody targeting IL-1β, IL-6 or TNF-α significantly attenuated TDP-43 ACM-induced neuronal toxicity (Fig. 3h; IL-1β: 52.6 ± 2.1% in the TDP-43 ACM group versus 80.6 ± 1.4% in the TDP-43 ACM + anti-IL-1β antibody group; IL-6: 52.6 ± 2.1% in the TDP-43 ACM group versus 78.5 ± 1.5% in the TDP-43 ACM + anti-IL-6 antibody group; TNF-α: 52.6 ± 2.1% in the TDP-43 ACM group versus 78.8 ± 1.4% in the TDP-43 ACM + anti-TNF-α antibody group; all test groups were compared to the GFP ACM group). Moreover, GFP and GFP + PTP1Bi ACM supplemented with IL-1β, IL-6 and TNF-α proteins also resulted in neurotoxicity similar to that of TDP-43 ACM (Figure S6a; 100 ± 2.0% in the GFP ACM group versus 66 ± 3.0% in the GFP ACM + IL-1β + IL-6 + TNF-α protein group; 99 ± 2.3% in the GFP + PTP1Bi ACM group versus 63 ± 2.0% in the GFP + PTP1Bi ACM + IL-1β + IL-6 + TNF-α protein group). Furthermore, siRNA knockdown of *Il*-*1b*, *Il*-*6* and *Tnf*-*α* in TDP-43-overexpressing astrocytes dramatically attenuated TDP-43-induced neuronal toxicity and reduced secretion of proinflammatory cytokines (Figure S7a–d). These data indicate that the upregulation of proinflammatory cytokines such as IL-1β, IL-6 and TNF-α is an essential process in astrocytic TDP-43-induced neuronal toxicity.

### PTP1B inhibition suppresses astrocytic TDP-43-induced mitochondrial dysfunction and spine retraction in neurons

**Fig. 4:**
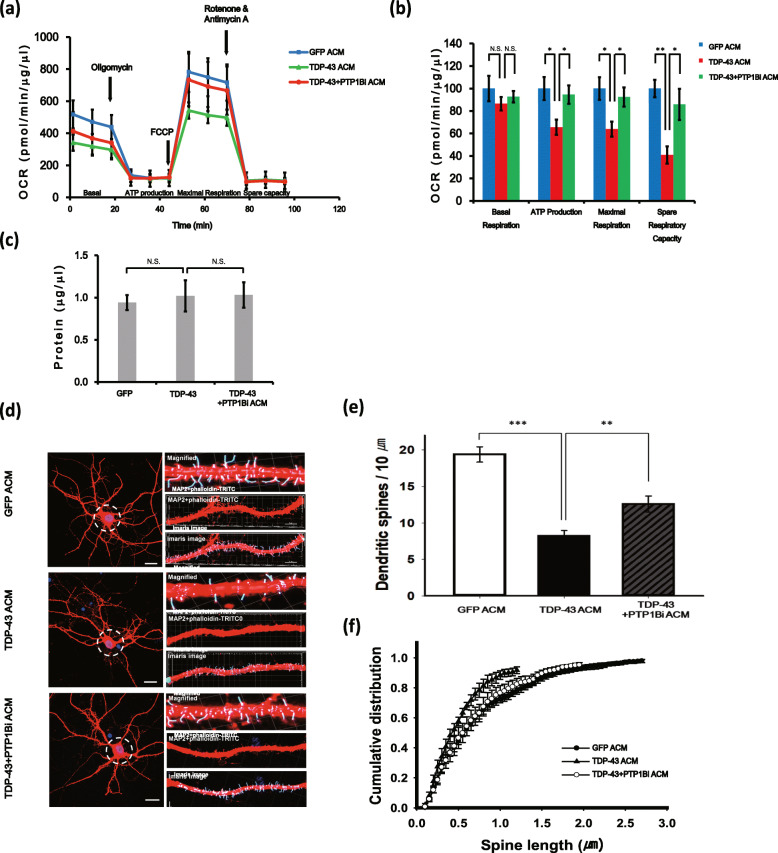
PTP1B inhibition suppresses TDP-43-induced mitochondrial dysfunction in astrocytes and spine retraction in primary cortical neurons. **a–c** Primary cortical neurons in XF24-well culture plates were stimulated with GFP ACM, TDP-43 ACM or TDP-43 + PTP1Bi ACM for 5 days. **a** Analysis of mitochondrial dysfunction in ACM-treated cells was performed by a Seahorse XF analyser to detect the basal OCR, ATP production, maximum reserve and respiratory capacity. The OCR was normalized to the total protein concentration (OD). **b** Quantification of the OCR, ATP production, maximum reserve, and respiratory capacity as a percentage of the basal values. Data are presented as the mean ± SEM. *p < 0.05; **p < 0.005; and *N*.*S*. not significant (one-way ANOVA). **c** The protein concentrations of cell lysates in each ACM-treated group were measured by a BCA protein assay kit. Data are presented as the mean ± SD. *N*.*S*. not significant (one-way ANOVA). **d** Representative confocal images of each ACM-treated group. Dendritic spines in the dotted circle, which is 20 μm radius from the centre of nucleus, were not analysed (panel left). Scale bars, 20 μm. Representative confocal (phalloidin-TRITC, red and anti-Map2-Alexa 594, red) and filament tracing Imaris images from randomly selected dendritic spines of each ACM-treated neuron group (right panel). Scale bars, 5 μm. The magnified image in the right panels shows filament tracing of the analysed spine. Scale bars, 2 μm. **e** Dendritic spine density was defined as the number of spines per 50 μm of dendrite length in the Imaris image of the right panel of Fig. 4d, which shows the average number of dendritic spines for 10 μm. Fifteen dendrites were randomly selected from four separate cortical neuron cultures per GFP ACM, TDP-43 ACM and TDP-43+PTP1Bi ACM-treated cortical neuron group. **p < 0.005, ***p < 0.001 (one-way ANOVA). **f** Cumulative distribution curves of spine length from cortical neurons as determined by the Kolmogorov-Smirnov (KS) test: mean spine length of GFP ACM vs. TDP-43 ACM and TDP-43 ACM vs. TDP-43 + PTP1Bi ACM. *p* = 0.027 and *p* = 0.609, respectively

Recent studies have suggested that mitochondrial dysfunction is a critical factor for many neurodegenerative diseases, including AD, PD and ALS [46–48]. Moreover, previous studies have indicated that TDP-43 is linked to mitochondrial dysfunction and abnormalities of mitochondrial dynamics [49–52]. Therefore, TDP-43-induced mitochondrial defects could be a key feature of disease pathology. To investigate whether TDP-43 overexpression in astrocytes could induce mitochondrial dysfunction in neurons, we measured the cellular OCR using a Seahorse XF24 Extracellular Flux Analyzer and a mitochondrial stress test kit (Seahorse Bioscience) in ACM-treated primary cortical neurons. The OCR is an indicator of mitochondrial respiration. OCR measurement in cultured neurons using electron transport chain-regulating agents such as oligomycin, FCCP and rotenone/antimycin A allows for the analysis of mitochondrial respiratory parameters, including basal respiration, ATP production, maximal respiration and spare respiratory capacity. The basal mitochondrial respiration was not significantly affected by TDP-43 ACM treatment (Fig. 4a, b; 86.4 ± 6.0% in the TDP-43 ACM group versus 92.7 ± 5.0% in the TDP-43 + PTP1Bi ACM group; all test groups were compared to the *Gfp*-transfected + DMSO-treated ACM group). However, ATP production, maximal respiration and spare respiratory capacity were markedly decreased by TDP-43 ACM treatment in primary cortical neurons (Fig. 4a, b). Consistent with the cell viability assay results, TDP-43-induced neuronal mitochondrial dysfunction in astrocytes was greatly ameliorated by PTP1B inhibition (Fig. 4a, b, ATP production: 65.6 ± 6.8% in the TDP-43 ACM group versus 94.6 ± 8.2% in the TDP-43 + PTP1Bi ACM group; maximal respiration capacity: 63.9 ± 6.6% in the TDP-43 ACM group versus 92.4 ± 8.5% in the TDP-43 + PTP1Bi ACM group; spare respiratory capacity: 40.8 ± 7.7% in the TDP-43 ACM group versus 85.8 ± 13.8% in the TDP-43 + PTP1Bi ACM group; all test groups were compared to the *Gfp*-transfected + DMSO-treated ACM group). Additionally, OCR values were normalized for each group according to total cellular protein concentration (Fig. 4c).

Mitochondrial functions associate with various neuronal diseases and locally modulate the formation of growth cones to affect the direction and rate of neurite growth [49, 53, 54]. Moreover, TDP-43 affects the local translation of dendritic mRNAs, which contributes to synaptic plasticity [55]. Therefore, we analysed neuronal morphology changes by TDP-43-induced neurodegeneration in astrocytes. TDP-43 ACM-treated neurons showed a significant reduction in spine density and length compared to those of GFP ACM-treated neurons (Fig. 4d–f). Quantitative analysis data of spine density and length were significantly decreased by treatment with the TDP-43 ACM compared to the GFP ACM in cortical neurons, whereas both spine density and length were significantly recovered in the TDP-43 + PTP1Bi ACM group compared to TDP-43 ACM group (Fig. 4d–f). Taken together, these data indicate that PTP1B inhibition mitigates several neurodegenerative phenotypes caused by TDP-43-induced alterations in astrocytes.

### PTP1B inhibition ameliorates astrocytic TDP-43-induced neuronal toxicity and mitochondrial dysfunction in motor neuron-like cells

**Fig. 5:**
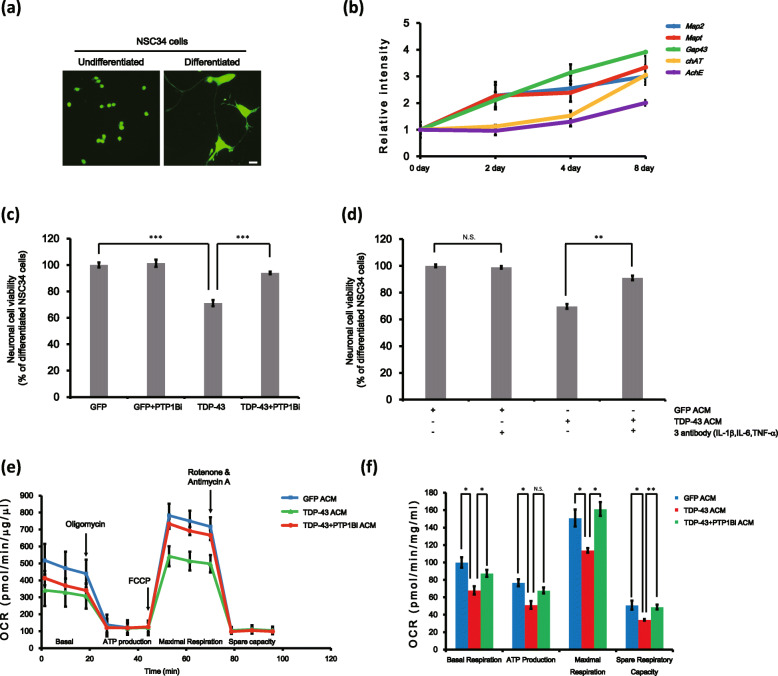
PTP1B inhibition attenuates astrocytic TDP-43-induced toxicity and mitochondrial dysfunction in differentiated NSC-34 motor neuron-like cells. **a**–**f** NSC-34 cells were treated with 1 μM RA for 8 days and then were analysed. **a** CMFDA staining of undifferentiated or differentiated NSC-34 cells at 8 days. Scale bars, 100 μm. **b** Real-time PCR results of motor neuronal marker genes in undifferentiated or differentiated NSC-34 cells at the indicated time points. Data are presented as the mean ± SD. *Gapdh* was used for normalization. **c** Differentiated NSC-34 cells were stimulated with GFP ACM, TDP-43 ACM or TDP-43+ PTP1Bi ACM for 4 days. Cell viability was analysed by CCK-8 assay. ***p < 0.005 (one-way ANOVA). **d** Differentiated NSC-34 cells stimulated with TDP-43 ACM were treated with an IL-1β antibody (50 ng/ml), an IL-6 antibody (50 ng/ml) and a TNF-α antibody (100 ng/ml) for 4 days and then were subjected to CCK-8 assays. TDP-43 ACM-induced toxicity was rescued by IL-1β, IL-6 and TNF-α antibody treatment. Data are presented as the mean ± SD. *p < 0.05; **p < 0.005 (one-way ANOVA). **e, f** Differentiated NSC-34 cells in XF24-well culture plates were stimulated with GFP ACM, TDP-43 ACM or TDP-43 + PTP1Bi ACM for 4 days. **e** Mitochondrial dysfunction analysis of ACM-treated cells was performed with a Seahorse XF analyser to detect the basal OCR, ATP production, maximum reserve and respiratory capacity. The OCR was normalized to total protein concentration. **f** Quantification of the OCR, ATP production, maximum reserve and respiratory capacity as a percentage of the basal values. Data are presented as the mean ± SEM. *p < 0.05; **p < 0.005; and *N*.*S*. not significant (one-way ANOVA)

We next investigated whether PTP1B inhibition also mitigates astrocytic TDP-43-induced neuronal toxicity in motor neuron-like cells. To do this, we induced the differentiation process in NSC-34 cells. Previous studies indicate that differentiated NSC-34 cells show motor neuron-like properties, such as neurite extension and the expression of specific motor neuron markers [34, 56, 57]. Differentiated NSC-34 cells showed motor neuron-like morphology and markedly increased transcription of motor neuron markers *Map2*, *Mapt*, *Gap43*, *chAT* and *AchE* (Fig. 5a, b). Similar to the results of the primary cortical neurons, the cellular toxicity of TDP-43 ACM in differentiated NSC-34 cells was significantly attenuated by PTP1B inhibition (Fig. 5c; 80 ± 3.6% in the TDP-43 ACM group versus 90 ± 1.3% in the TDP-43 + PTP1Bi ACM group; all test groups were compared to the *Gfp*-transfected + DMSO-treated ACM group). In addition, treatment with antibodies targeting IL-1β, IL-6 and TNF-α dramatically attenuated the cellular toxicity of TDP-43 ACM in differentiated NSC-34 cells (Fig. 5d; 69 ± 1.7% in the TDP-43 ACM group versus 90 ± 1.6% in the TDP-43 ACM + anti-IL-1β + anti-IL-6 + anti-TNF-α antibody group; all test groups were compared to the GFP ACM group).

To further confirm that IL-1β, IL-6 and TNF-α proteins are essential components of astrocytic TDP-43-induced neurotoxicity, the effect of IL-1β, IL-6 and TNF-α treatment was examined in differentiated NSC-34 cells. Similar to the situation observed with TDP-43 ACM treatment, GFP or GFP + PTP1Bi ACM supplemented IL-1β, IL-6 and TNF-α caused neurotoxicity (Figure S6b; 100 ± 3.1% in the GFP ACM group versus 44 ± 2.0% in the GFP ACM + IL-1β + IL-6 + TNF-α protein group; 98 ± 1.1% in the GFP + PTP1Bi ACM group versus 46 ± 0.7% in the GFP + PTP1Bi ACM + IL-1β + IL-6 + TNF-α protein group).

To investigate whether TDP-43 overexpression in astrocytes could induce mitochondrial dysfunction in motor neuron-like cells, we measured the cellular OCR in ACM-treated differentiated NSC-34 cells. The basal mitochondrial respiration, ATP production, maximal respiration and spare respiratory capacity were markedly decreased by TDP-43 ACM treatment in differentiated NSC-34 cells (Fig. 5e, f). Similar to the results observed in primary cortical neurons, TDP-43-induced mitochondrial dysfunction in astrocytes was greatly mitigated by PTP1B inhibition in differentiated NSC-34 cells (Fig. 5e, f; basal mitochondrial respiration: 67.9 ± 4.7% in the TDP-43 ACM group versus 87.4 ± 3.9% in the TDP-43 + PTP1Bi ACM group; ATP production: 51.2 ± 4.4% in the TDP-43 ACM group versus 67.8 ± 3.4% in the TDP-43 + PTP1Bi ACM group; maximal respiration capacity: 113.8 ± 2.5% in the TDP-43 ACM group versus 161.2 ± 7.8% in the TDP-43 + PTP1Bi ACM group; spare respiratory capacity: 34.2 ± 0.9% in the TDP-43 ACM group versus 48.9 ± 2.7% in the TDP-43 + PTP1Bi ACM group; all test groups were compared to *Gfp*-transfected + DMSO-treated ACM group). Moreover, GFP ACM or GFP + PTP1Bi ACM supplemented with IL-1β, IL-6 and TNF-α caused mitochondrial dysfunction in differentiated NSC-34 cells (Figure S6c). These results suggest that PTP1B also regulates TDP-43-induced astrocyte death in motor neuron-like cells.

### Inflammation and neuronal toxicity induced by glial TDP-43 are mitigated by *Ptp1b* downregulation in *Drosophila*

**Fig. 6:**
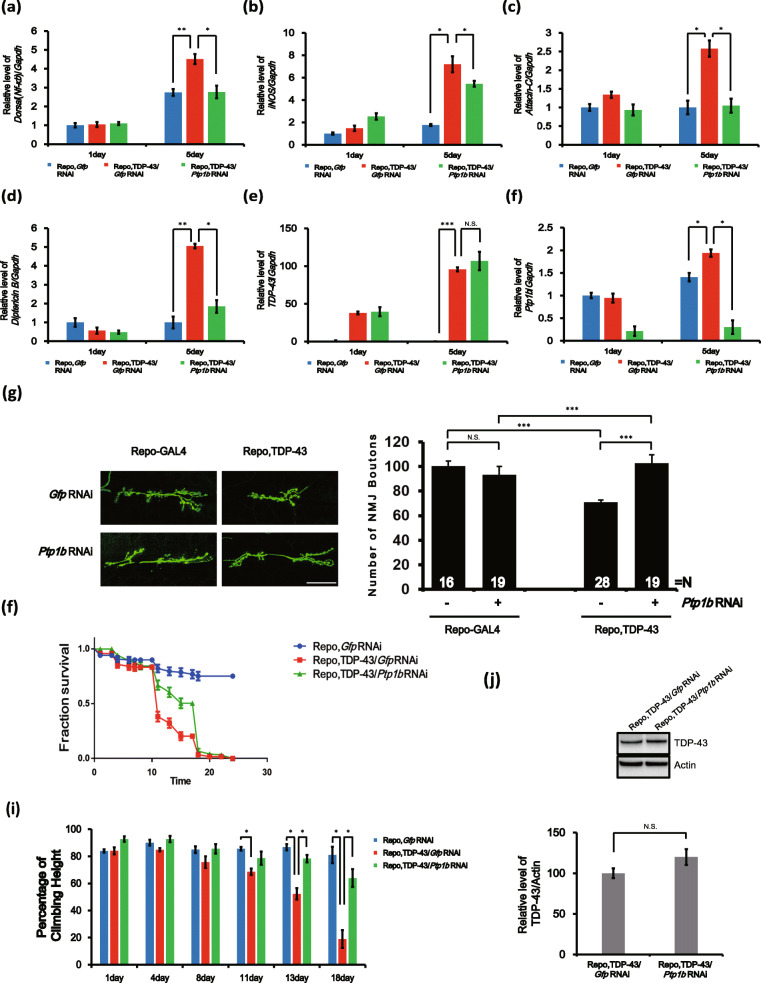
The inflammation and neuronal toxicity effects induced by glial TDP-43 are mitigated by *Ptp61f* downregulation in *Drosophila.* **a**, **b** Levels of *Dorsal* (*Nf-κb*) and *iNOS* mRNA from fly head lysates of control or TDP-43 glial-expressing transgenic flies were analysed by real-time PCR. *Gapdh* was used for normalization. Data are presented as the mean ± SD. *p < 0.05; **p < 0.005 (one-way ANOVA). **c**, **d** Levels of *Attacin-C* and *Diptericin B* mRNA from fly head lysates of control or TDP-43-expressing glial transgenic flies were analysed by real-time PCR. *Gapdh* was used for normalization. Data are presented as the mean ± SD. *p < 0.05; **p < 0.005 (one-way ANOVA). **e**, **f** Levels of *Tdp-43* and *Ptp1b* mRNA from fly head lysates of control or TDP-43-expressing glial transgenic flies were analysed by real-time PCR. *Gapdh* was used for normalization. Data are presented as the mean ± SD. *p < 0.05; ***p < 0.001; *N*.*S*. not significant (one-way ANOVA). **g** Immunostaining of third instar larval NMJs under wild-type (Repo-Gal4/+) and TDP-43-overexpressing (Repo-Gal4/TDP-43) conditions. Labelling of NMJs to determine their morphology in larva was conducted with anti-horseradish peroxidase (green). Scale bars, 50 μm. Data are presented as the mean ± SEM. ***p < 0.001; *N*.*S*. not significant (one-way ANOVA). **h** Survival rate of flies treated with Repo-Gal4/GFP RNAi, Repo,TDP-43/GFP RNAi or Repo,TDP-43/PTP1B RNAi at the indicated time points. Data are presented as the mean ± SEM. **i** Climbing activity of flies treated with Repo-Gal4/GFP RNAi, Repo, TDP-43/GFP RNAi or Repo,TDP-43/PTP1B RNAi at the indicated time points. Data are presented as the mean ± SEM. *p < 0.05 (one-way ANOVA). **j** Immunoblot analysis of TDP-43 proteins from head lysates of control or TDP-43 glial transgenic flies. Actin was used for normalization. Data are presented as the mean ± SD. *N*.*S*. not significant (Student’s *t* test). **a**–**j** Genotypes: GFP is *Repo-Gal4/UAS-Egfp RNAi*^*VALIUM20-EGFP.shRNA.4*^, GFP + PTP1B RNAi is *Repo-Gal4/UAS- Ptp61f RNAi* ^*HMS00421*^, TDP-43 is *Repo-Gal4/UAS-TDP-43/UAS-Egfp RNAi*^*VALIUM20-EGFP.shRNA.4*^ and TDP-43+PTP1B RNAi is *Repo-Gal4/UAS-TDP-43/UAS-Ptp61f RNAi* ^*HMS00421*^

Previous studies indicate that the NF-κB pathway is a key mechanism for regulating inflammation in *Drosophila* [58]. We wondered whether the modification of PTP1B expression affected TDP-43-induced inflammation in vivo, so we used a *Drosophila* ALS model that expressed human TDP-43 in all glial cells. Overexpression of TDP-43 in glial cells significantly increased *Dorsal* (*Nf*-*κb*) and *iNOS* levels after 5 days (Fig. 6a, b). The downregulation of *Ptp1b* in *Drosophila* glial cells suppressed the levels of inflammatory genes induced by TDP-43 expression (Fig. 6a, b) without changing the expression level of TDP-43 (Fig. 6e). The knockdown efficiency of *Ptp61f* RNAi (the *Drosophila* homologue of PTP1B) was high, as *Ptp61f* levels were low (Fig. 6f). In addition, the activation of NF-κΒ leads to the production of antimicrobial peptides (AMPs), such as Attacin, Diptericin and Cecropin [59–61]. We examined the effects of *Ptp1b* and TDP-43 expression on TDP-43-induced AMP genes (Figs. 6c, d and S8a–f). TDP-43-expressing flies showed markedly increased *Attacin*-*C* and *Diptericin B* expression. *Attacin*-*C* and *diptericin B* expression was significantly suppressed by the downregulation of *Ptp1b* in *Drosophila* (Figs. 6c, d and S8a–f). These results suggest that the downregulation of *Ptp1b* in the *Drosophila* glial system is sufficient for attenuating TDP-43-induced inflammation.

To investigate the relevance of PTP1B for TDP-43-induced neurotoxicity, we used a model of *Drosophila* expressing human TDP-43 in all types of glial cells, including astrocyte-like glia. Although TDP-43 can induce inflammation, it is still not clear whether the inhibition of PTP1B is implicated in TDP-43-mediated neurotoxicity in *Drosophil*a. The *Drosophila* NMJ is a powerful biological system for studying synaptic defects in neurodegenerative diseases (Lu et al. 2011; Lee et al. 2012; McGurk et al. 2015). A recent study showed that the overexpression of TDP-43 in motor neurons leads to a significant disruption of NMJ morphology (Coyne et al. 2014). In another study, it was shown that disrupted expression of TDP-43 at NMJs impairs BMP signalling (Deshpande et al. 2016). However, glial toxicity caused by TDP-43 has not been well-characterized in NMJs. We found that glial expression of TDP-43 at larval NMJs resulted in an ~ 30% reduction in the bouton number. This effect was rescued by knocking down *Ptp61f* expression with an RNAi (Fig. 6g). These results provide strong evidence for a genetic role of PTP1B in regulating neuronal toxicity caused by TDP-43 overexpression in glia.

Previously, we revealed that flies expressing TDP-43 showed a markedly reduced lifespan and climbing ability compared to those of controls [11]. We also examined the effects of *Ptp1b* and TDP-43 expression on TDP-43-induced lifespan reduction and climbing dysfunction. The TDP-43-expressing flies showed a markedly reduced lifespan and climbing ability (Fig. 6h, i) without any change in the expression level of TDP-43 (Fig. 6j). The lifespan and climbing defects induced by TDP-43 were significantly rescued by the downregulation of *Ptp61f* in *Drosophila* (Fig. 6h, i). At 18 days of age, flies expressing TDP-43 in their glia showed a markedly reduced climbing ability compared to control animals (18.9 ± 6.6% in the Repo, TDP-43/GFP RNAi versus 81.0 ± 6.0% in the Repo-Gal4/GFP RNAi). This climbing deficit was greatly attenuated by knockdown of *Ptp61f* (18.9 ± 6.6% in the Repo,TDP-43/GFP RNAi versus 63.9 ± 6.5% in the Repo,TDP-43/PTP1B RNAi), with the flies also exhibiting a recovered motility. Knockdown of *Ptp61f* alone had no effect on climbing ability or lifespan (data not shown). These results indicate that PTP1B inhibition mitigates neuronal toxicity caused by pan-glial TDP-43-induced inflammation in flies.

**Fig. 7:**
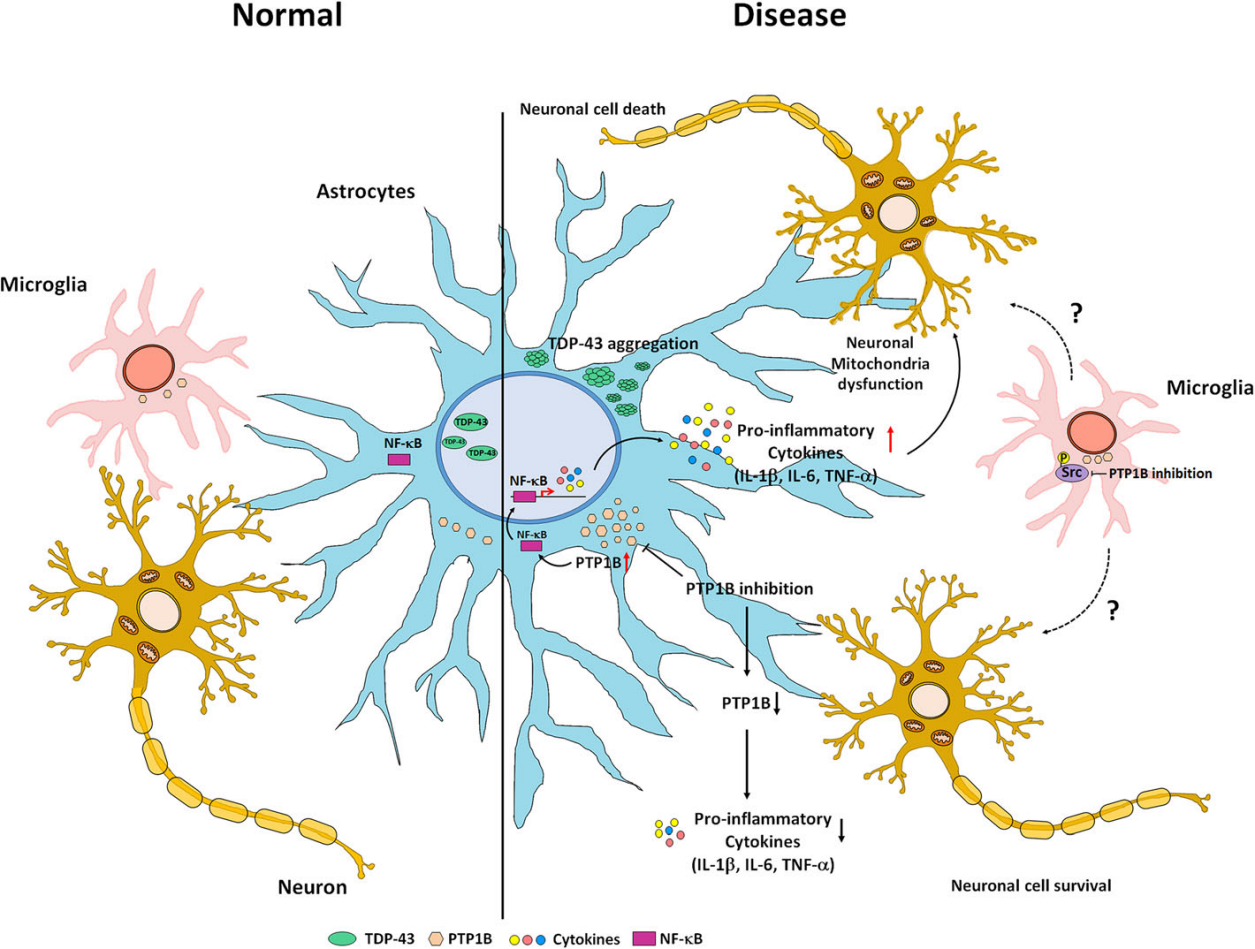
Schematic representation of the effect of PTP1B inhibition on the neurotoxicity of glial TDP-43 overexpression. In the disease state, an increase in cytoplasmic TDP-43 aggregation in astrocytes leads to the accumulation of PTP1B. PTP1B upregulation activates NF-κB p65 and induces the translocation of NF-κB to the nucleus. Nuclear NF-κB upregulates the transcriptional level of proinflammatory genes. This transcriptional activation increases the secretion of proinflammatory cytokines and chemokines, such as IL-1β, IL-6 and TNF-α. Subsequently, the extensive secretion of proinflammatory cytokines and chemokines leads to neuronal death. Thus, PTP1B inhibition mitigates neurodegeneration caused by TDP-43-induced inflammation in astrocytes

Taken together, our data support a model whereby TDP-43 in astrocytes causes neurodegeneration at least in part by inducing the inflammatory response via PTP1B (Fig. 7). We found that TDP-43-induced PTP1B upregulation eventually leads to activation of the NF-κB pathway in astrocytes. Activation of the NF-κB pathway also increases the secretion of inflammatory cytokines, such as IL-1β, IL-6 and TNF-α. Consequently, proinflammatory activation of astrocytes induces mitochondrial dysfunction, spine retraction, and cell death in nearby neurons.

## Discussion

We demonstrated for the first time that PTP1B is a major regulator of the TDP-43-induced inflammatory response in astrocytes. It is already known that the TDP-43 protein initiates the proinflammatory cascade in microglia [41, 62]; however, its role in astrocytes was previously unknown. Our present study shows changes in the inflammatory and toxic functions of astrocytes due to TDP-43, and it provides a mechanistic understanding of the role of these alterations in exacerbating neurodegeneration.

Previous studies have shown that astrocytes isolated from ALS patients are toxic to mouse embryonic stem cell-derived motor neurons in coculture [63]. More recently, Qian et al. demonstrated that astrocytes differentiated from induced pluripotent stem cells of ALS patients cause movement deficits and neurodegeneration in mice following their transplantation into the spinal cords of SCID mice [64]. Although the underlying molecular mechanism of astrocyte-mediated neurotoxicity in ALS is not well understood, accumulating evidence suggests that astrocytes are implicated in neurodegeneration in ALS.

Recent studies have demonstrated that PTP1B positively modulates neuroinflammation via the NF-κB pathway in microglia [25, 65]. However, the role of PTP1B in astrocytes is largely unknown. Here, we found that PTP1B inhibition effectively attenuates the TDP-43-induced inflammatory response in astrocytes. Similar to what is observed in microglia, PTP1B regulates the inflammatory response via the NF-κB pathway (Fig. 2a–c). NF-κB activation is observed in the astrocytes of ALS patients [66]. In addition, Kia et al. showed that astrocytes expressing ALS-linked mutant FUS were toxic to motor neurons and that mutant FUS-expressing astrocyte-induced neurotoxicity is mediated by the NF-κB pathway [45]. Notably, TDP-43 directly binds to the p65 subunit of NF-κB and acts as a coactivator of the NF-κB pathway in BV-2 microglial cells [67]. These findings suggest that activation of NF-κB in astrocytes is implicated in neurodegeneration in ALS and that this activation might be induced by TDP-43 accumulation.

The secretion of proinflammatory cytokines is known to be associated with glia-mediated neurotoxicity. Previous studies have found that administration of IL-1β in the rat brain dramatically enhances neuronal damage induced by ischaemia and excitotoxicity [68, 69]. In this study, we found that the secretion of IL-1β, IL-6 and TNF-α in astrocytes was elevated by overexpression of TDP-43 (Fig. 2d–f). Interestingly, these cytokines were previously shown to be significantly elevated in the peripheral blood of ALS patients [70]. Moreover, a recent meta-analysis revealed that TNF-α was significantly increased in the cerebrospinal fluid (CSF) of ALS patients [71]. To determine whether the increase in inflammatory cytokine levels by astrocytic TDP-43 is related to neurotoxicity, we treated cells with neutralizing antibodies targeting IL-1β, IL-6 and TNF-α. Accordingly, we found that TDP-43-overexpressing astrocyte-induced neurotoxicity is attenuated by treatment with these neutralizing antibodies (Fig. 3f–h). Our findings, together with others, suggest that TDP-43 upregulation in astrocytes may be linked to elevated levels of proinflammatory cytokines and that these cytokines are mediators of neuronal death in ALS.

Mitochondrial dysfunction in affected neurons is a common feature of ALS [72]. However, although numerous studies have shown that neuronal TDP-43 accumulation causes abnormalities in mitochondrial morphology, dynamics and function with in vivo and in vitro models [8, 52, 73–75], it is unknown whether the accumulation of TDP-43 in glia can induce neuronal mitochondrial impairment. In this study, seahorse analysis of mitochondrial bioenergetics in cortical neurons treated with TDP-43 ACM revealed that TDP-43 ACM significantly impaired the maximal respiration rate and the ATP-linked respiration rate. Moreover, PTP1B inhibition greatly attenuated TDP-43 ACM-induced mitochondrial dysfunction (Fig. 4a, b). Collectively, the findings from our study demonstrated that TDP-43 accumulation in astrocytes drives neuronal mitochondrial defects via the PTP1B-mediated inflammatory response. To study the effects of TDP-43 on motor neuron-like cell lines, we investigated the toxicity and mitochondrial dysfunction induced by TDP-43 in differentiated NSC-34 cells. NSC-34 is a hybrid cell line produced by the fusion of neuroblastoma/spinal cord neurons, and it is often used as a bona fide cellular model to investigate the physiological mechanism of ALS [76]. Our data confirmed that PTP1B inhibition mitigated cytotoxicity and mitochondrial dysfunction induced by TDP-43 ACM in differentiated NCS-34 cells (Fig. 5c–f).

NF-κB-mediated immune responses in glia are well conserved in *Drosophila* [77]. Furthermore, in *Drosophila*, genetic suppression of the NF-κB pathway (Imd/Relish) greatly attenuated the neuronal TDP-43-induced shortening of lifespan [78]. Although some studies indicate that glial TDP-43 expression causes premature death, motility deficits and larval NMJ defects [79–81], it is still unclear whether glial TDP-43 can induce neuroinflammation in *Drosophila*. In this study, our data in *Drosophila* showed that the expression of inflammatory genes (*Dorsal* (*Nf*-*κb*), *iNOS*, *Attacin*-*C*, *Diptericin B*) is greatly increased by pan-glial TDP-43 expression (Fig. 6a–d). Importantly, our data also showed that genetic knockdown of fly PTP1B effectively restored the upregulation of those inflammatory genes. Consistent with previous results, pan-glial expression of TDP-43 in *Drosophila* causes larval NMJ defects, a shortened lifespan and climbing defects, and these pathologic phenotypes were significantly suppressed by knockdown of fly PTP1B (Fig. 6g–i). These findings suggest that inhibition of PTP1B mitigates glial TDP-43-induced neurotoxicity in vivo.

Three glial cell types are mainly associated with major neurons in the *Drosophila* central nervous system (CNS): astrocyte-like, ensheathing and cortex glia. These 3 types of glia share the function of mammalian astrocytes. They surround neuronal cell bodies and proximal neurites, are coupled to the vasculature, and associate closely with synapses [82]. To regulate target genes in all glial cells, we used the repo-GAL4 driver, which expresses GAL4 protein in all types of glial cells except for midline glia. Similar to the results of mammalian astrocytes, glial TDP-43-induced inflammation and neurotoxicity are significantly attenuated by knockdown of *Ptp1b*. Thus, we believe that the fly model data using repo-GAL4 further confirm the results from the mouse astrocyte primary culture experiments. However, we cannot rule out the possibility that the functions of fly glial cells are different from those of mouse astrocytes, which may have affected these results. Thus, further in-depth studies are warranted to elucidate how PTP1B regulates glial TDP-43-induced neurodegeneration in *Drosophila*.

## Conclusion

Our data highlight the therapeutic potential of PTP1B in treatment of ALS. Since PTP1B is a well-known therapeutic target for diabetes and obesity, numerous PTP1B inhibitors have already been developed [83]. Therefore, analysing the effects of the previously developed PTP1B inhibitors on astrocytic TDP-43-induced neurotoxicity may help to identify promising therapeutic agents.

## Supplementary information

**Additional file 1: Figure S1**. Purity of cultured primary mouse cortical neurons and astrocytes*.* (a) Primary cortical neuron (*upper*) or astrocyte (*lower*) enriched cultures were stained with antibodies for neuron, astrocyte and microglia markers. Triple immunostaining of MAP2 (neuron; red), GFAP (astrocytes; green), and Iba-1 (microglia; pink) in primary neuronal cells at DIV 7 and astrocytes at DIV 21. DAPI staining was used to determine the number of cells. Scale bar, 200 μm. *n*=248 cells (primary cortical neuron culture), *n*=447 cells (primary astrocyte culture). **Figure S2**. Insoluble TDP-43 protein was significantly increased in TDP-43-overexpressing primary astrocytes. (a) Immunoblot analysis of TDP-43 protein in the insoluble and soluble fractions of *TDP-43-Gfp*-transfected astrocytes. The immunoblot results from 3 independent experiments were normalized to those of tubulin. **Figure S3**. Transfection of the control plasmid did not affect the viability of astrocytes. (a) Astrocytes were treated with the Lipofectamine only or with a GFP expression DNA vector + Lipofectamine mixture for 3 days; then, CCK-8 assays were performed. Data are presented as the mean ± SD of 3 independent experiments. N.S., not significant (Student’s *t*-test). **Figure S4**. PTP1B and proinflammatory genes are upregulated by TDP-43 overexpression in primary astrocytes. (a-e) *TDP-43-Gfp*-transfected astrocytes were treated with a PTP1B inhibitor (PTP1Bi, 5 μM) for 1 day, and then real-time PCR was performed. *18S rRNA* was used as a normalization gene for real-time PCR data. PTP1B inhibition greatly attenuated TDP-43-induced inflammatory upregulation. Quantification data for *Il-1b* (a), *Il-6* (b), *Lcn2* (c), *iNos* (d), and *Nf-κb*. (e) Quantification data are presented as the mean ± SD from 3 independent real-time PCR experiments. *p<0.05; **p<0.005; and ***p<0.001 (one-way ANOVA with Bonferroni’s multiple comparison test). (f-j) Astrocytes were cotransfected with *TDP-43* expression construct and a control siRNA or mouse *Ptp1b* siRNA for 3 days, and then FACS of *Gfp*-transfected live cells was performed. These cells were allowed to acclimate for 1 day and then were subjected to real-time PCR experiments. The TDP-43-induced upregulation of inflammatory gene transcription was attenuated by PTP1B downregulation. *18S rRNA* was used as a normalization gene for RT-PCR. Quantification data for *Il-1b* (f), *Il-6* (g), *Lcn2* (h), *iNos* (i), and *Nf-κb*. (j) All data are presented as the mean ± SD from 3 independent real-time PCR experiments. *p<0.05; **p<0.005 (one-way ANOVA with Bonferroni’s multiple comparison test). **Figure S5**. ACM from astrocytes treated with a PTP1B inhibitor does not affect the viability of mouse cortical neurons. (a) Primary cortical neurons were treated with DMSO ACM or PTP1Bi ACM for 5 days and then were subjected to CMFDA staining. CMFDA-positive neurons were counted under a fluorescence microscope. The percentage of CMFDA-positive cells was quantified (*lower*). Data are presented as the mean ± SD of 3 independent experiments. N.S., not significant (Student’s *t*-test). Scale bars, 20 μm. **Figure S6**. The secretion of proinflammatory cytokines such as IL-1β, IL-6, and TNF-α mediates astrocytic TDP-43-induced neuronal toxicity and mitochondrial dysfunction. Primary cortical neurons (a) and differentiated NSC-34 motor neurons (b) were stimulated with GFP ACM or GFP + PTP1Bi ACM supplemented with IL-1β protein (50 ng/ml), IL-6 protein (50 ng/ml), and TNF-α protein (10 ng/ml) for 4 days, and then they were subjected to CCK-8 assays. Similar to what was observed with TDP-43 ACM treatment, GFP or GFP + PTP1Bi ACM supplemented with IL-1β, IL-6, and TNF-α caused neurotoxicity. Data are presented as the mean ± SD of 3 independent experiments. **p<0.005 (Student’s *t*-test). (c) Differentiated NSC-34 motor neurons stimulated with GFP ACM or GFP + PTP1Bi ACM supplemented with IL-1β protein (50 ng/ml), IL-6 protein (50 ng/ml), and TNF-α protein (10 ng/ml) for 4 days and then subjected to mitochondrial dysfunction analysis. Mitochondrial dysfunction analysis of ACM-treated cells was assessed through the detection of basal OCR, ATP production, maximum reserve and respiratory capacity by a Seahorse XF analyser. The oxygen consumption rate (OCR) was normalized to the total protein concentration (OD). Quantification of the OCR, ATP production, maximum reserve and respiratory capacity as a percentage of the basal values. Data are presented as the mean ± SEM of 3 independent experiments. *p<0.05; **p<0.005; and ***p<0.001 (one-way ANOVA with Bonferroni’s multiple comparison test). **Figure S7**. Knockdown of *Il-1b, Il-6,* and *Tnf-*α mitigated astrocytic TDP-43-induced neuronal toxicity. (a-c) Astrocytes were cotransfected with the *TDP-43-Gfp* expression construct and control siRNA, *Il-1b* siRNA, *Il-6* siRNA, or *Tnf-*α siRNA, and after 3 days the ACM were harvested. The concentrations of secreted cytokines (IL-1β, IL-6, and TNF-α) in the GFP ACM and TDP-43 ACM groups were measured by ELISA. TDP-43-induced secretion of cytokines (IL-1β, IL-6, and TNF-α) was significantly suppressed by the downregulation of IL-1β (a), IL-6 (b), or TNF-α. (c). Data are presented as the mean ± SD. *p<0.05; **p<0.005; and ***p<0.001 (one-way ANOVA with Bonferroni’s multiple comparison test). (d) Primary cortical neurons stimulated with TDP-43ACM from astrocytes were treated with *Il-1b* siRNA, *Il-6* siRNA, and *Tnf-a* siRNA for 5 days, and then they were subjected to CCK-8 assays. TDP-43 ACM-induced toxicity was rescued by siRNA knockdown of proinflammatory cytokine genes. Data are presented as the mean ± SD of 3 independent experiments. *p<0.05; **p<0.005; and ***p<0.001 (one-way ANOVA with Bonferroni’s multiple comparison test). **Figure S8**. Inflammation induced by glial TDP-43 is mitigated by *Drosophila Ptp1b* downregulation. (a-f) Levels of *Dorsal* (*Nf-κb*), *iNos*, *Attacin-C*, *Diptericin B*, *TDP-43* and *Ptp1b* mRNA from fly head lysates of control or TDP-43-expressing glial transgenic flies were analysed by real-time PCR. *18S rRNA* was used as a normalization gene for real-time PCR. TDP-43-induced expression of genes involved in inflammation and genes that are downstream of NF-κB was significantly suppressed by the downregulation of PTP1B. Quantification data of *Dorsal* (*Nf-κb*) (a), *iNos* (b), *Attacin-C* (c), *Diptericin B* (d), *TDP-43* (e), and *Ptp1b* (f) mRNA transcript levels are presented as the mean ± SD from 3 independent real-time PCR experiments. *18S rRNA* was used for normalization. *p<0.05; **p<0.005; and ***p<0.001 (one-way ANOVA with Bonferroni’s multiple comparison test).

## Data Availability

The authors declare the data and material availability in this manuscript.

## Abbreviations

ALS: Amyotrophic lateral sclerosis
TDP-43: Transactive response DNA binding protein of 43kDa
FTLD-TDP: Frontotemporal lobar degeneration with TDP-43
hnRNP: Heterogeneous nuclear ribonucleoprotein
WT: Wild type
AD: Alzheimer’s disease
FTLD: Frontotemporal lobal degeneration
PTP1B: Protein-tyrosine phosphatase 1B
DMSO: Dimethyl sulfoxide
RA: All-*trans* retinoic acid
ACM: Astrocyte-conditioned media
CMFDA: Chloromethylfluorescein diacetate
IL-1β: Interleukin 1 beta
IL-6: Interleukin 6
TNF-α: Tumour necrosis factor alpha
FCCP: Carbonyl cyanide 4-(trifluoromethoxy) phenylhydrazone
OCR: Oxygen consumption rate
AMPs: Antimicrobial peptides
NMJ: Neuromuscular junction
CSF: Cerebrospinal fluid
CNS: Central nervous system
