# Supplementary material for: The overexpression of TDP-43 in astrocytes causes neurodegeneration via a PTP1B-mediated inflammatory response
Source: J Neuroinflammation. 2020 Oct 14;17:299. doi: 10.1186/s12974-020-01963-6 (PMC7556969; doi:10.1186/s12974-020-01963-6)
Supplement: Supplementary file 1 — Additional file 1: Figure S1. Purity of cultured primary mouse cortical neurons and astrocytes. (a) Primary cortical neuron (upper) or astrocyte (lower) enriched cultures were stained with antibodies for neuron, astrocyte and microglia markers. Triple immunostaining of MAP2 (neuron; red), GFAP (astrocytes; green), and Iba-1 (microglia; pink) in primary neuronal cells at DIV 7 and astrocytes at DIV 21. DAPI staining was used to determine the number of cells. Scale bar, 200 μm. n=248 cells (primary cortical neuron culture), n=447 cells (primary astrocyte culture). Figure S2. Insoluble TDP-43 protein was significantly increased in TDP-43-overexpressing primary astrocytes. (a) Immunoblot analysis of TDP-43 protein in the insoluble and soluble fractions of TDP-43-Gfp-transfected astrocytes. The immunoblot results from 3 independent experiments were normalized to those of tubulin. Figure S3. Transfection of the control plasmid did not affect the viability of astrocytes. (a) Astrocytes were treated with the Lipofectamine only or with a GFP expression DNA vector + Lipofectamine mixture for 3 days; then, CCK-8 assays were performed. Data are presented as the mean ± SD of 3 independent experiments. N.S., not significant (Student’s t-test). Figure S4. PTP1B and proinflammatory genes are upregulated by TDP-43 overexpression in primary astrocytes. (a-e) TDP-43-Gfp-transfected astrocytes were treated with a PTP1B inhibitor (PTP1Bi, 5 μM) for 1 day, and then real-time PCR was performed. 18S rRNA was used as a normalization gene for real-time PCR data. PTP1B inhibition greatly attenuated TDP-43-induced inflammatory upregulation. Quantification data for Il-1b (a), Il-6 (b), Lcn2 (c), iNos (d), and Nf-κb. (e) Quantification data are presented as the mean ± SD from 3 independent real-time PCR experiments. *p<0.05; **p<0.005; and ***p<0.001 (one-way ANOVA with Bonferroni’s multiple comparison test). (f-j) Astrocytes were cotransfected with TDP-43 expression construct and a control siRN [file 12974_2020_1963_MOESM1_ESM.docx]

**Supplementary Information**

**The overexpression of TDP-43 in astrocytes causes neurodegeneration via a PTP1B-mediated inflammatory response**

**Short running title: TDP-43 induced inflammation in astrocytes**

Shinrye Lee¹^,*^, Seyeon Kim^1,2,*^, Ha-Young Kang^3^, Hye Ryeong Lim^7^, Younghwi Kwon^1, 2^, Myungjin Jo^1^, Yu-Mi Jeon^1^, Sang Ryong Kim^4,5^, Kiyoung Kim^6^, Chang Man Ha^7^, Seongsoo Lee^3,#^ and Hyung-Jun Kim¹^,#^

^1^Dementia Research Group, Korea Brain Research Institute (KBRI), Daegu, South Korea, 41062

^2^Department of Brain & Cognitive Sciences, DGIST, Daegu, South Korea, 42988

^3^Gwangju Center, Korea Basic Science Institute (KBSI), Gwangju, South Korea, 61886

^4^School of Life Sciences, BK21 Plus KNU Creative BioResearch Group, Institute of Life Science & Biotechnology, Kyungpook National University, Daegu, South Korea, 41566

^5^Brain Science and Engineering Institute, Kyungpook National University, Daegu, South Korea, 41944

^6^Department of Medical Biotechnology, Soonchunhyang University, Asan, South Korea, 31538

^7^Research Division and Brain Research Core Facilities, Korea Brain Research Institute (KBRI), Daegu, South Korea, 41062

^*^These authors contributed equally to this work.

^#^Correspondence and requests for materials should be addressed to S Lee and H-J Kim

(Email: soolee@kbsi.re.kr , kijang1@kbri.re.kr)

**Supplementary methods**

**Antibodies** The following antibodies were used for immunocytochemistry (ICC): mouse anti-MAP2 (1:500; M4403) [Sigma]; rabbit anti-GFAP (1:500; Z033429) [Dako]; and goat anti-Iba-1 (1:500; ab5076) [Abcam].

**Preparation of soluble and insoluble cell extracts** *TDP-43*-transfected astrocytes (15 × 10^4^ cells/well) were grown in 6-well plates and were homogenized in RIPA buffer with protease and phosphatase inhibitor cocktails (Roche). Soluble and insoluble fractions in 1% Triton X-100 were obtained by centrifugation at 100,000 × g for 30 min at 4 °C. Supernatants containing the soluble fractions were harvested, and the pellets for insoluble fractions were solubilized in 2% SDS detergent Cell Lysis buffer (Cell Signaling). After sonication, the cell lysates were mixed with 4x Bolt LDS Sample buffer and 10x Bolt Sample Reducing Agent buffer and then were boiled at 95 °C for 5 min.

**IL-1b, IL-6, or TNF-α protein-supplemented ACM** To generate IL-1b, IL-6, or TNF-α protein-supplemented ACM, *Gfp*-transfected live primary astrocytes (15 × 10^4^ cells/well) obtained via FACS were allowed to acclimate for 24 hr in DMEM. *Gfp*-transfected cells were treated with a PTP1B inhibitor (PTP1Bi, 5 μM), and then IL-1β protein (50 ng/ml), IL-6 protein (50 ng/ml), or TNF-α protein (10 ng/ml) were added to the media for 24 hr. The cells were then washed twice with PBS and cultured in fresh DMEM for an additional 24 hr. The ACM was then collected, centrifuged at 200 × g for 10 min to remove cell debris, and stored at -80 °C until further analysis.

**siRNA transfection** Primary astrocytes in 6-well plates (40 × 10^4^ cells/well) were cotransfected with a *pCMV6-AC-TDP-43-Gfp* vector and control siRNA (Dharmacon; D-001810-10), mouse *Il-1b* siRNA (Dharmacon; L-060344-00), mouse *Il-6* siRNA (Dharmacon; L-043739-00), or mouse *Tnf-a* siRNA (Dharmacon; L-042302-00) using Lipofectamine 3000 reagent (Invitrogen) or Lipofectamine RNAiMAX reagent (Invitrogen), and then the cells were incubated for 3 days. The downregulation of target protein expression in the transfected cells was confirmed by immunoblot analysis. At 72 hr posttransfection, transfected cells were subjected to FACS of *Gfp*-transfected live cells, which were then fixed or harvested for further analyses.

**Statistical analyses** Data were analysed by Student’s *t*-test (Vassar Stats, [www.vassarstats.net](http://www.vassarastats.net)) or one-way ANOVA test depending on comparison variables with post hoc analysis as indicated (GraphPad Prism Software). Differences were considered significant when *p*<0.05 and are indicated as follows: **p*<0.05; ***p*<0.005; ****p*<0.001; and *n.s.,* not significant.

**Supplementary Figure Legends**

**Fig. S1** Purity of cultured primary mouse cortical neurons and astrocytes*.* (a) Primary cortical neuron (*upper*) or astrocyte (*lower*) enriched cultures were stained with antibodies for neuron, astrocyte and microglia markers. Triple immunostaining of MAP2 (neuron; red), GFAP (astrocytes; green), and Iba-1 (microglia; pink) in primary neuronal cells at DIV 7 and astrocytes at DIV 21. DAPI staining was used to determine the number of cells. Scale bar, 200 µm. *n*=248 cells (primary cortical neuron culture), *n*=447 cells (primary astrocyte culture).

**Fig. S2** Insoluble TDP-43 protein was significantly increased in TDP-43-overexpressing primary astrocytes. (a) Immunoblot analysis of TDP-43 protein in the insoluble and soluble fractions of *TDP-43-Gfp*-transfected astrocytes. The immunoblot results from 3 independent experiments were normalized to those of tubulin.

**Fig. S3** Transfection of the control plasmid did not affect the viability of astrocytes. (a) Astrocytes were treated with the Lipofectamine only or with a GFP expression DNA vector + Lipofectamine mixture for 3 days; then, CCK-8 assays were performed. Data are presented as the mean ± SD of 3 independent experiments. N.S., not significant (Student’s *t*-test).

**Fig. S4** PTP1B and proinflammatory genes are upregulated by TDP-43 overexpression in primary astrocytes. (a-e) *TDP-43-Gfp*-transfected astrocytes were treated with a PTP1B inhibitor (PTP1Bi, 5 µM) for 1 day, and then real-time PCR was performed. *18S rRNA* was used as a normalization gene for real-time PCR data. PTP1B inhibition greatly attenuated TDP-43-induced inflammatory upregulation. Quantification data for *Il-1b* (a), *Il-6* (b), *Lcn2* (c), *iNos* (d), and *Nf-κb*. (e) Quantification data are presented as the mean ± SD from 3 independent real-time PCR experiments. **p*<0.05; ***p*<0.005; and ****p*<0.001 (one-way ANOVA with Bonferroni’s multiple comparison test). (f-j) Astrocytes were cotransfected with *TDP-43* expression construct and a control siRNA or mouse *Ptp1b* siRNA for 3 days, and then FACS of *Gfp*-transfected live cells was performed. These cells were allowed to acclimate for 1 day and then were subjected to real-time PCR experiments. The TDP-43-induced upregulation of inflammatory gene transcription was attenuated by PTP1B downregulation. *18S rRNA* was used as a normalization gene for RT-PCR. Quantification data for *Il-1b* (f), *Il-6* (g), *Lcn2* (h), *iNos* (i), and *Nf-κb*. (j) All data are presented as the mean ± SD from 3 independent real-time PCR experiments. **p*<0.05; ***p*<0.005 (one-way ANOVA with Bonferroni’s multiple comparison test).

**Fig. S5** ACM from astrocytes treated with a PTP1B inhibitor does not affect the viability of mouse cortical neurons. (a) Primary cortical neurons were treated with DMSO ACM or PTP1Bi ACM for 5 days and then were subjected to CMFDA staining. CMFDA-positive neurons were counted under a fluorescence microscope. The percentage of CMFDA-positive cells was quantified (*lower*). Data are presented as the mean ± SD of 3 independent experiments. N.S., not significant (Student’s *t*-test). Scale bars, 20 µm.

**Fig. S6** The secretion of proinflammatory cytokines such as IL-1β, IL-6, and TNF-α mediates astrocytic TDP-43-induced neuronal toxicity and mitochondrial dysfunction. Primary cortical neurons (a) and differentiated NSC-34 motor neurons (b) were stimulated with GFP ACM or GFP + PTP1Bi ACM supplemented with IL-1β protein (50 ng/ml), IL-6 protein (50 ng/ml), and TNF-α protein (10 ng/ml) for 4 days, and then they were subjected to CCK-8 assays. Similar to what was observed with TDP-43 ACM treatment, GFP or GFP + PTP1Bi ACM supplemented with IL-1β, IL-6, and TNF-α caused neurotoxicity. Data are presented as the mean ± SD of 3 independent experiments. ***p*<0.005 (Student’s *t*-test). (c) Differentiated NSC-34 motor neurons stimulated with GFP ACM or GFP + PTP1Bi ACM supplemented with IL-1β protein (50 ng/ml), IL-6 protein (50 ng/ml), and TNF-α protein (10 ng/ml) for 4 days and then subjected to mitochondrial dysfunction analysis. Mitochondrial dysfunction analysis of ACM-treated cells was assessed through the detection of basal OCR, ATP production, maximum reserve and respiratory capacity by a Seahorse XF analyser. The oxygen consumption rate (OCR) was normalized to the total protein concentration (OD). Quantification of the OCR, ATP production, maximum reserve and respiratory capacity as a percentage of the basal values. Data are presented as the mean ± SEM of 3 independent experiments. **p*<0.05; ***p*<0.005; and ****p*<0.001 (one-way ANOVA with Bonferroni’s multiple comparison test).

**Fig. S7** Knockdown of *Il-1b, Il-6,* and *Tnf-*α mitigated astrocytic TDP-43-induced neuronal toxicity. (a-c) Astrocytes were cotransfected with the *TDP-43-Gfp* expression construct and control siRNA, *Il-1b* siRNA, *Il-6* siRNA, or *Tnf-*α siRNA, and after 3 days the ACM were harvested. The concentrations of secreted cytokines (IL-1β, IL-6, and TNF-α) in the GFP ACM and TDP-43 ACM groups were measured by ELISA. TDP-43-induced secretion of cytokines (IL-1β, IL-6, and TNF-α) was significantly suppressed by the downregulation of IL-1β (a), IL-6 (b), or TNF-α. (c). Data are presented as the mean ± SD. **p*<0.05; ***p*<0.005; and ****p*<0.001 (one-way ANOVA with Bonferroni’s multiple comparison test). (d) Primary cortical neurons stimulated with TDP-43ACM from astrocytes were treated with *Il-1b* siRNA, *Il-6* siRNA, and *Tnf-a* siRNA for 5 days, and then they were subjected to CCK-8 assays. TDP-43 ACM-induced toxicity was rescued by siRNA knockdown of proinflammatory cytokine genes. Data are presented as the mean ± SD of 3 independent experiments. **p*<0.05; ***p*<0.005; and ****p*<0.001 (one-way ANOVA with Bonferroni’s multiple comparison test).

**Fig. S8** Inflammation induced by glial TDP-43 is mitigated by *Drosophila Ptp1b* downregulation. (a-f) Levels of *Dorsal* (*Nf-κb*), *iNos*, *Attacin-C*, *Diptericin B*, *TDP-43* and *Ptp1b* mRNA from fly head lysates of control or TDP-43-expressing glial transgenic flies were analysed by real-time PCR. *18S rRNA* was used as a normalization gene for real-time PCR. TDP-43-induced expression of genes involved in inflammation and genes that are downstream of NF-κB was significantly suppressed by the downregulation of PTP1B. Quantification data of *Dorsal* (*Nf-κb*) (a), *iNos* (b), *Attacin-C* (c), *Diptericin B* (d), *TDP-43* (e), and *Ptp1b* (f) mRNA transcript levels are presented as the mean ± SD from 3 independent real-time PCR experiments. *18S rRNA* was used for normalization. **p*<0.05; ***p*<0.005; and ****p*<0.001 (one-way ANOVA with Bonferroni’s multiple comparison test).

**Supplementary Figures**

**Fig. S1** Purity of cultured primary mouse cortical neurons and astrocytes*.*

**
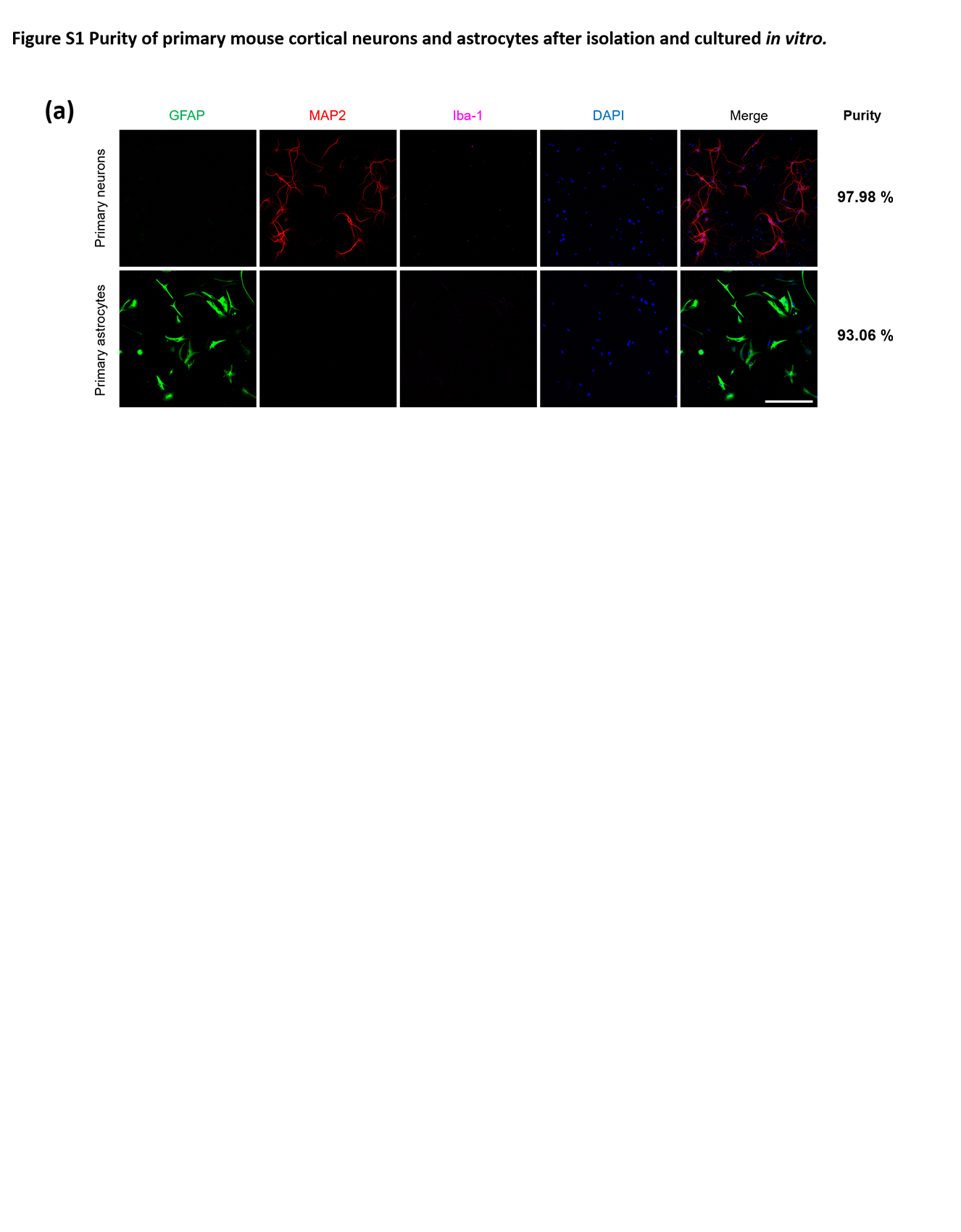
**

**Fig. S2** Insoluble TDP-43 protein was significantly increased in TDP-43-overexpressing primary astrocytes.


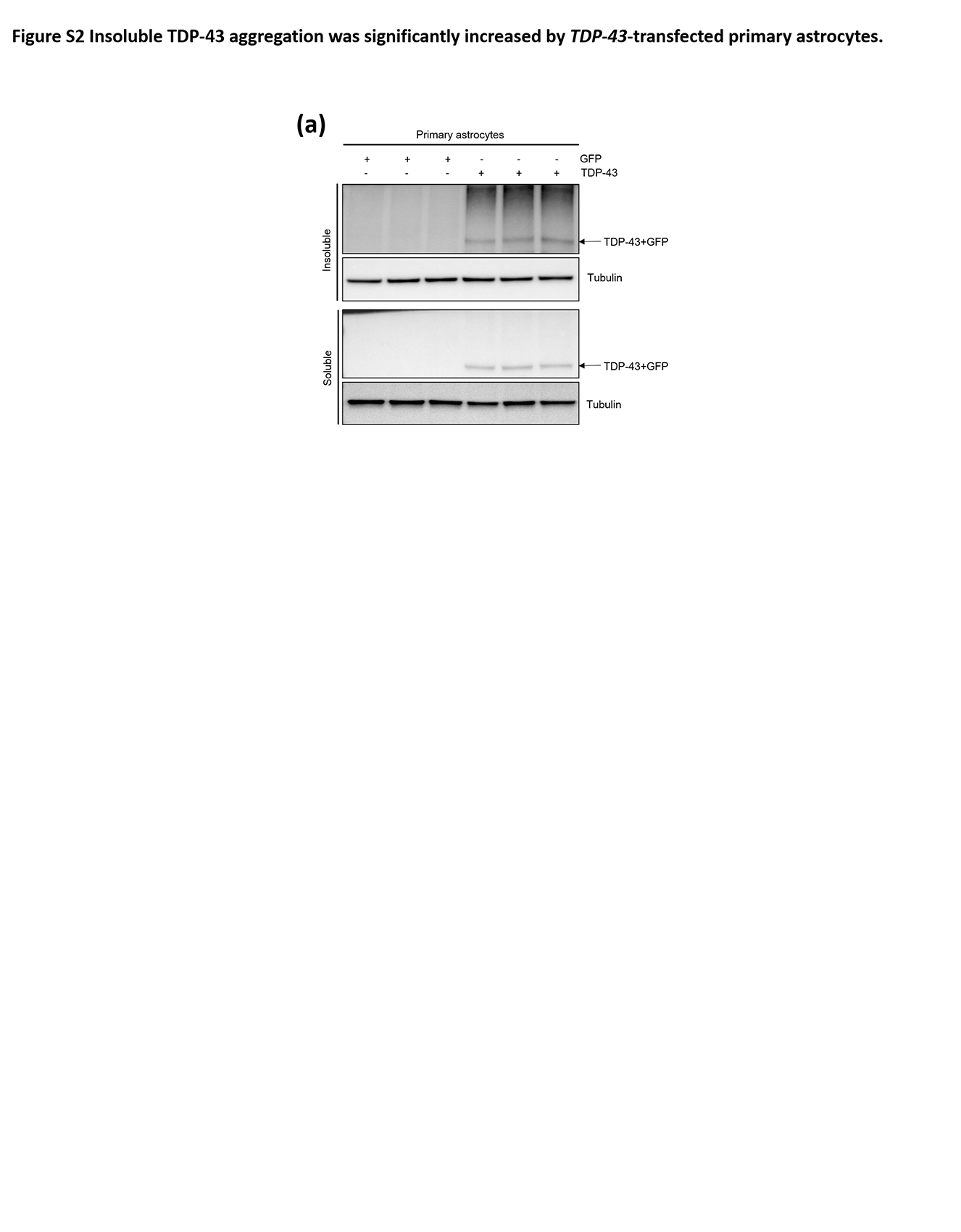


**Fig. S3** Transfection of control plasmid does not affect the cell viability of astrocytes.


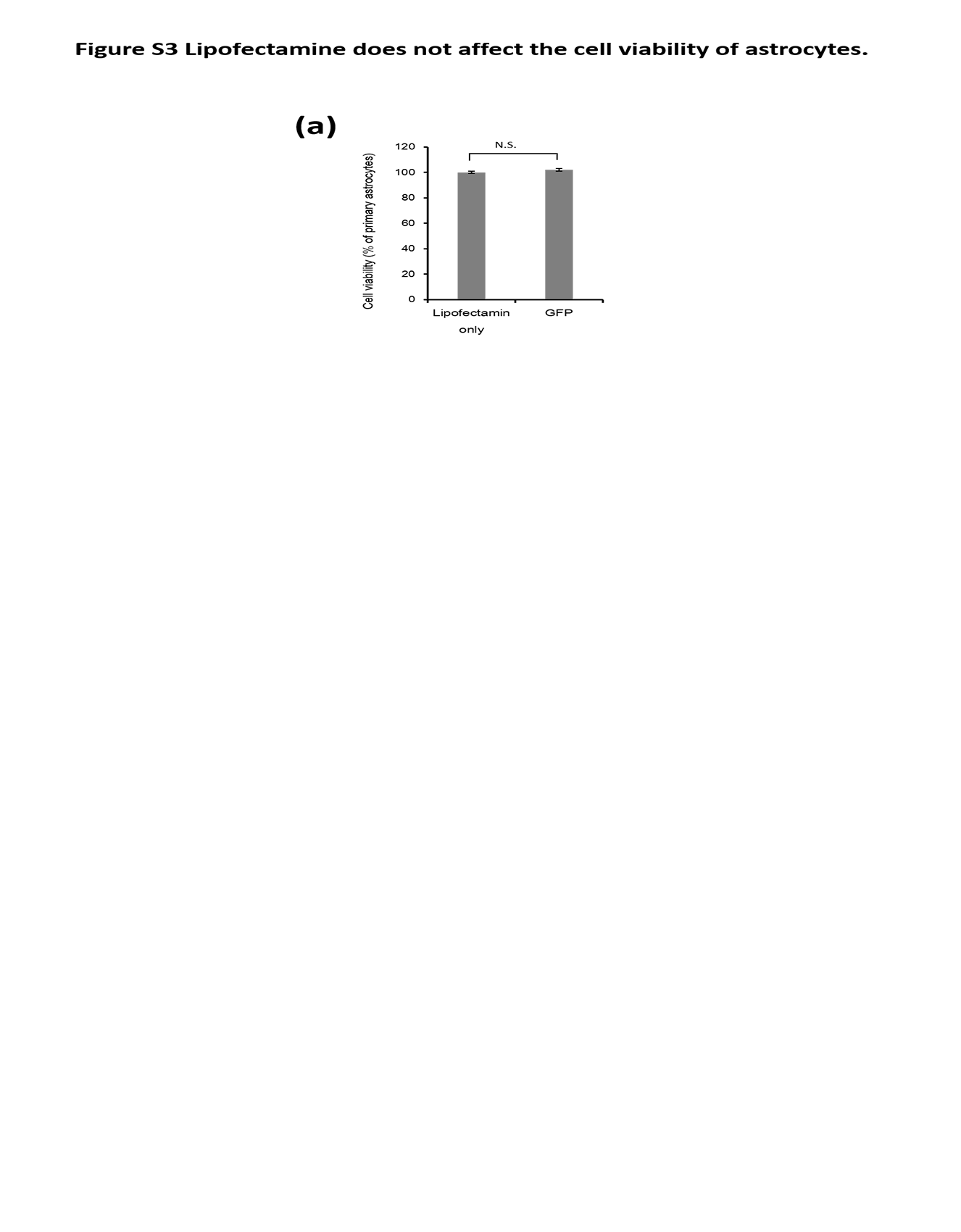


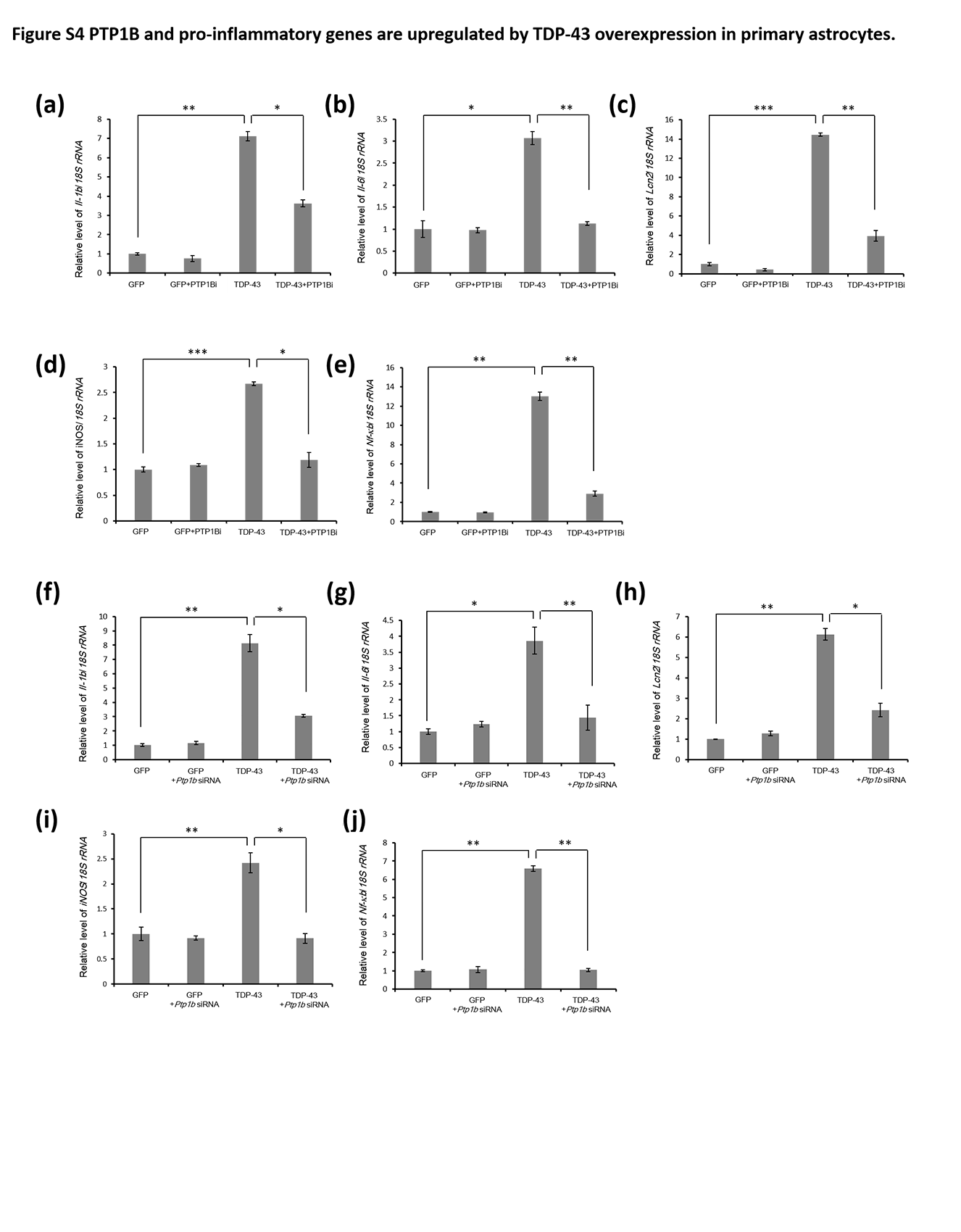
 **Fig. S4** PTP1B and pro-inflammatory genes are upregulated by TDP-43 overexpression in primary astrocytes.

**Fig. S5** ACM from astrocytes treated with PTP1B inhibitor does not affect the cell viability of mouse cortical neurons.


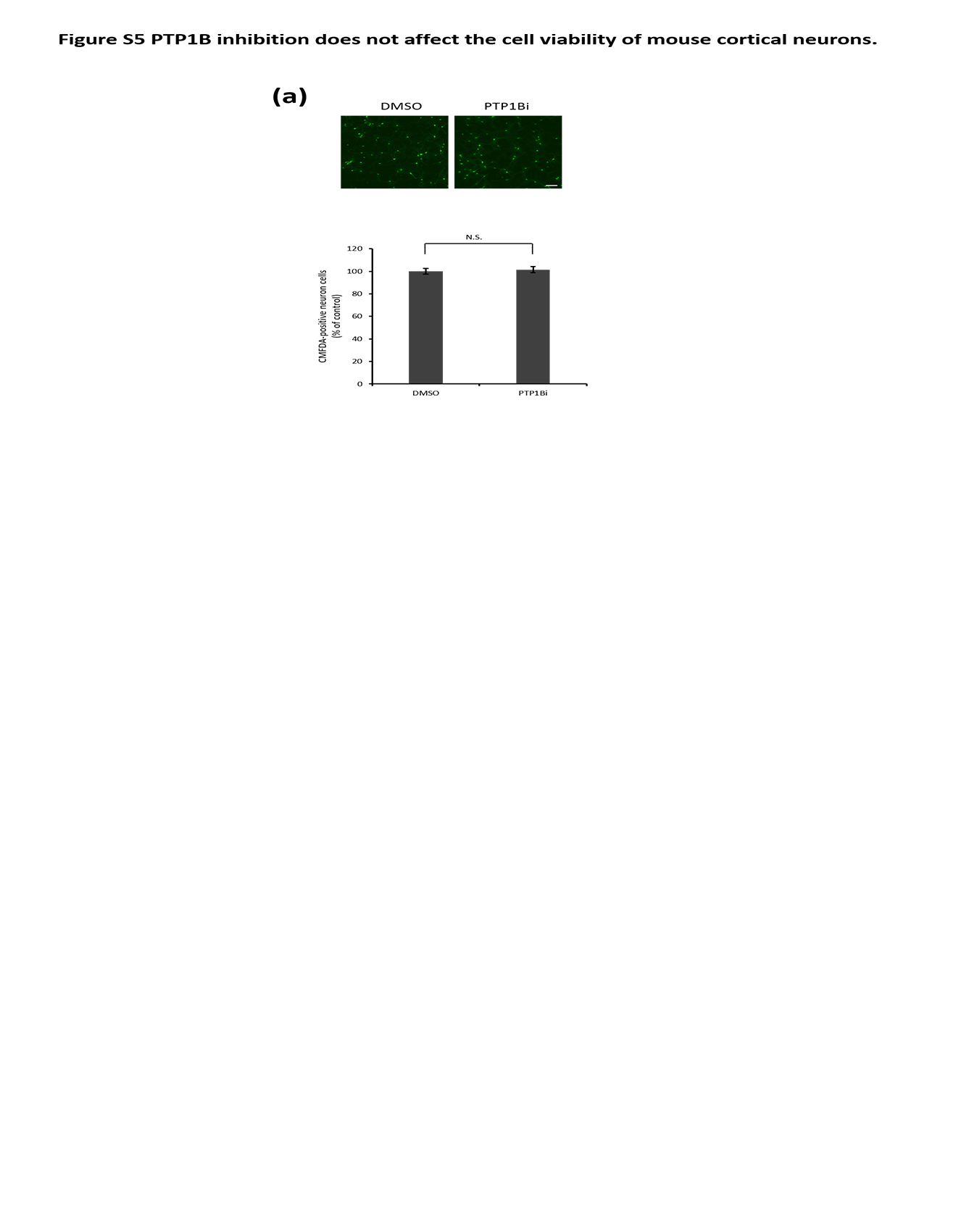


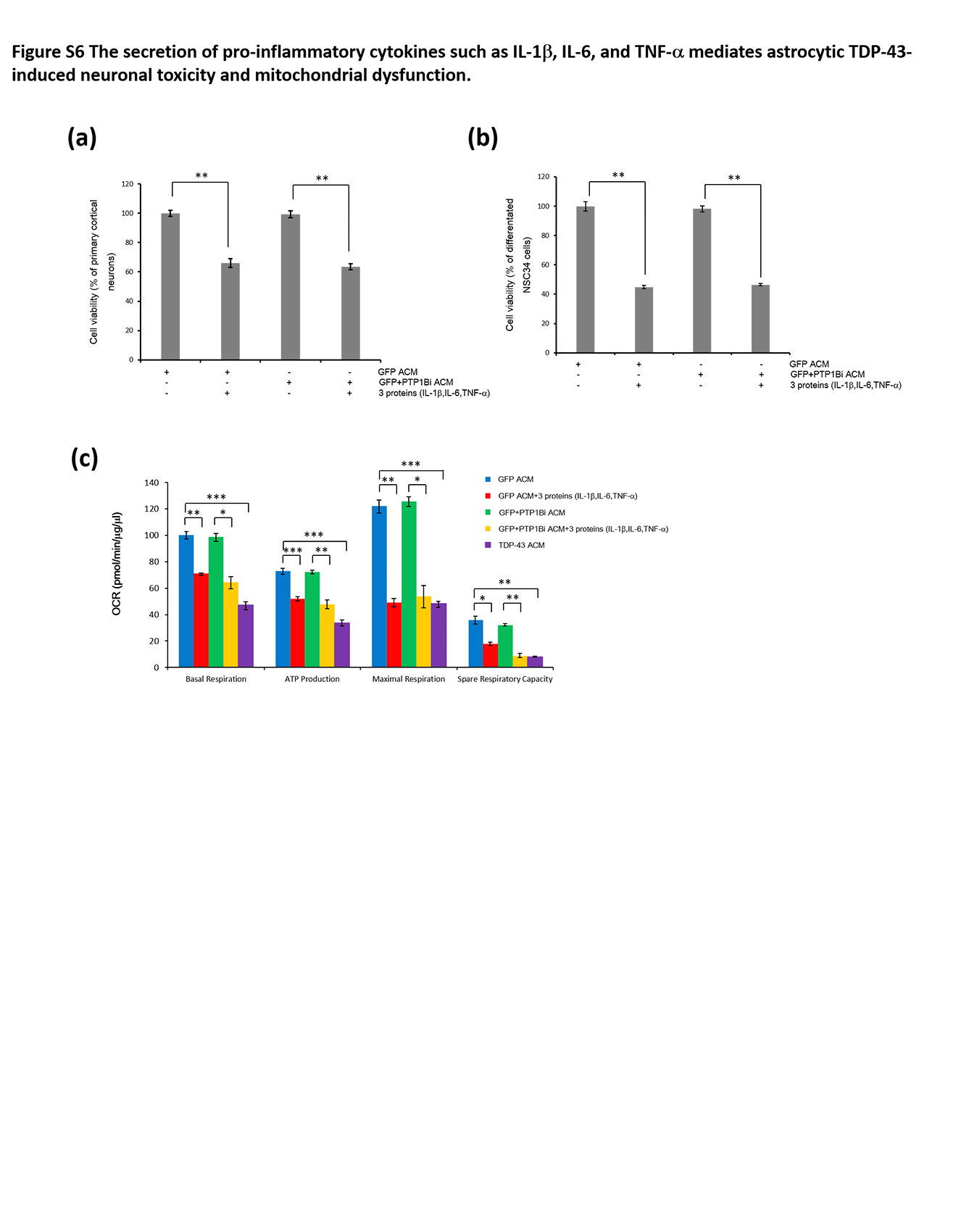
 **Fig. S6** The secretion of pro-inflammatory cytokines such as IL-1β, IL-6, and TNF-α mediates astrocytic TDP-43-induced neuronal toxicity and mitochondrial dysfunction.


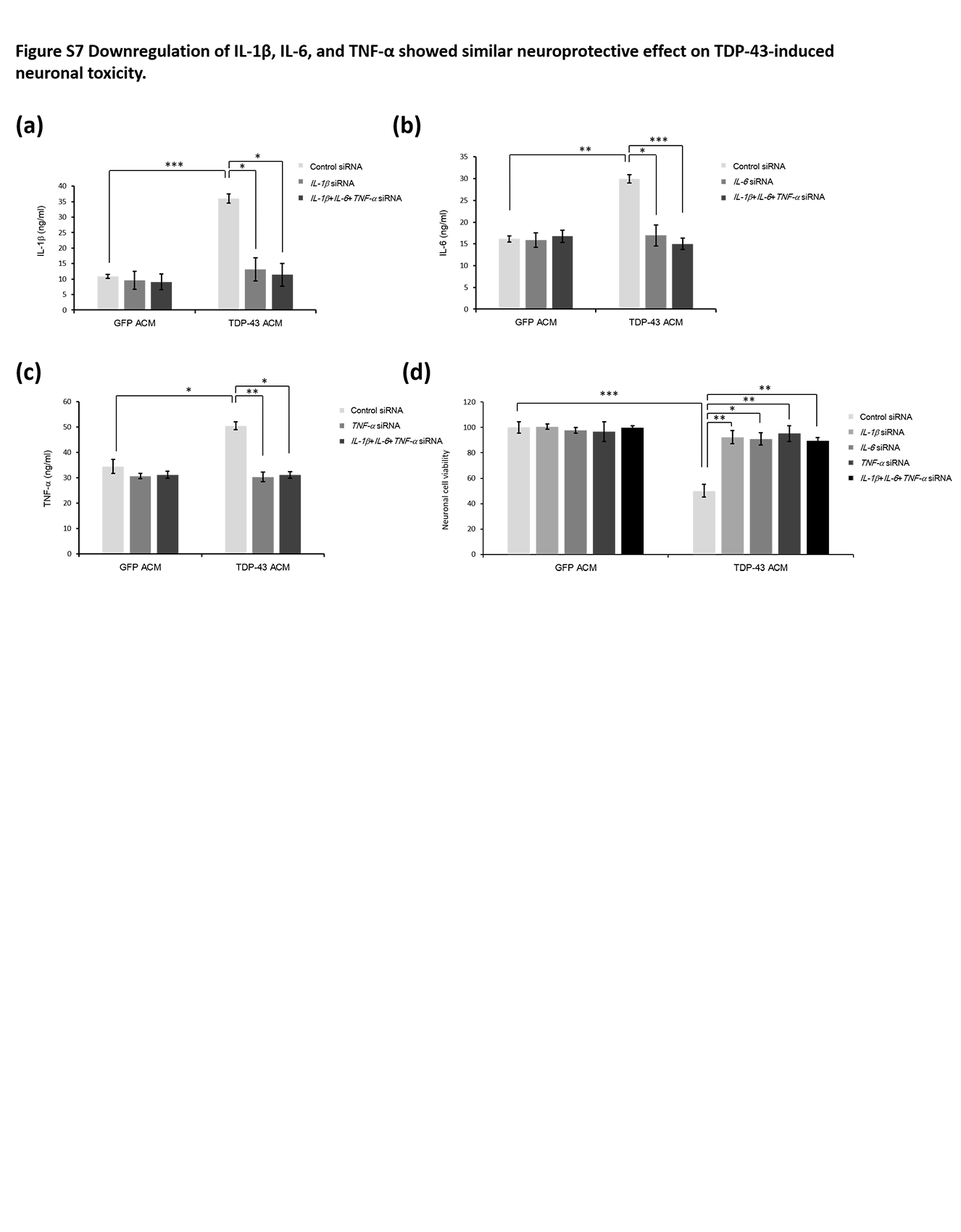
 **Fig. S7** Knockdown of *Il-1b, Il-6,* and *Tnf-a* mitigated astrocytic TDP-43-induced neuronal toxicity.


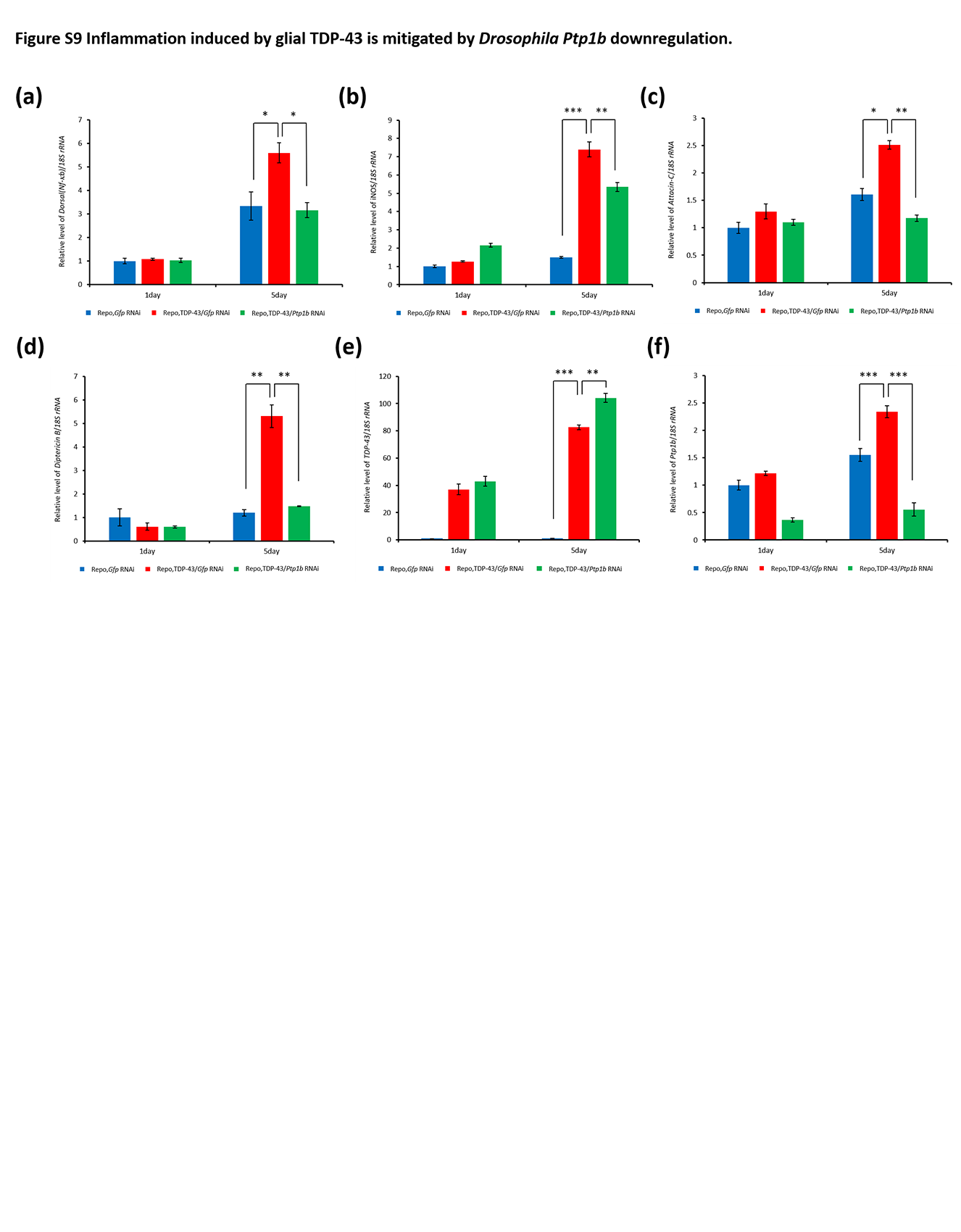
 **Fig. S8** Inflammation induced by glial TDP-43 is mitigated by *Drosophila Ptp1b* downregulation.
